# Supplementary material for: Gaps and opportunities for data systems and economics to support priority setting for climate-sensitive infectious diseases in sub-Saharan Africa: A rapid scoping review
Source: PLOS Glob Public Health. 2025 Jun 11;5(6):e0003814. doi: 10.1371/journal.pgph.0003814 (PMC12157337; doi:10.1371/journal.pgph.0003814)
Supplement: S1 Fig — Adapted from: World Health Organization. (2014). Ebola and Marburg virus disease epidemics: Preparedness, alert, control, and evaluation. https://www.who.int/publications-detail-redirect/WHO-HSE-PED-CED-2014.05 (DOCX) [file pgph.0003814.s001.docx]

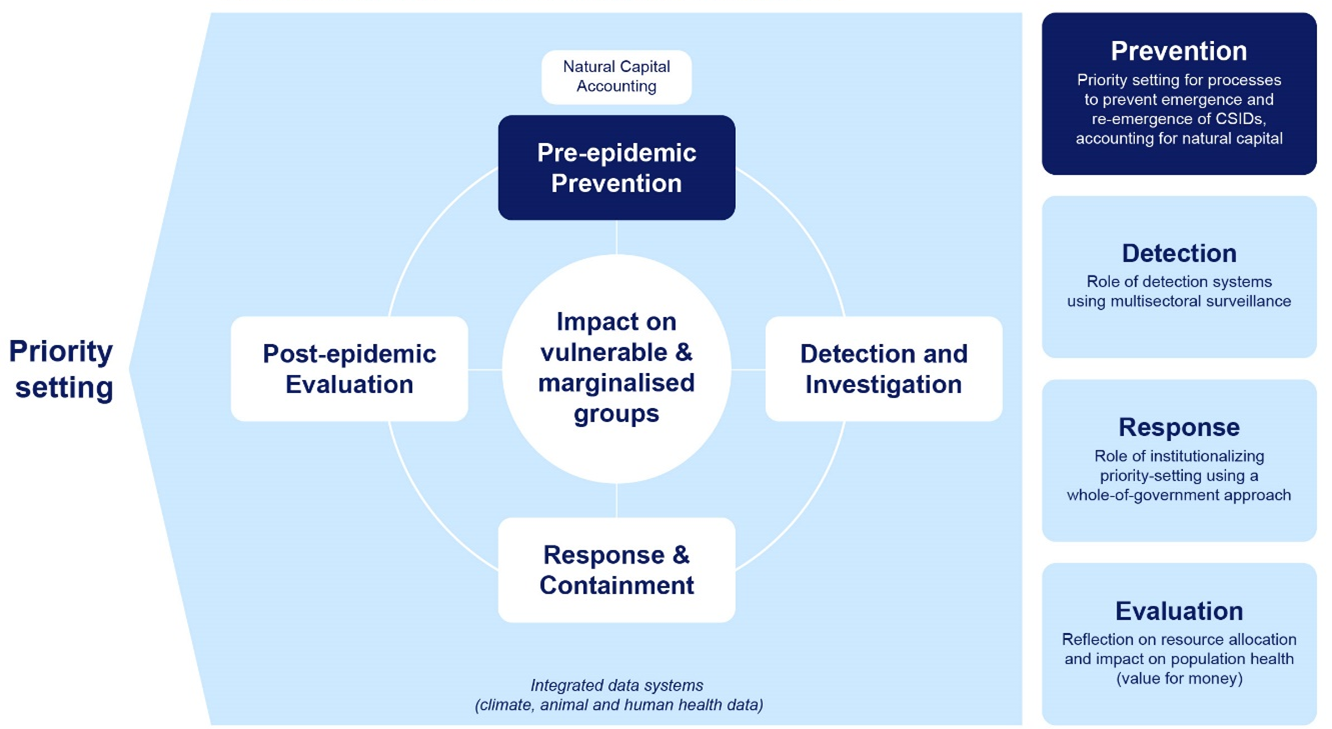


**S1 Fig. Pandemic Preparedness Framework for Climate Sensitive Infectious Disease in Africa. Adapted from: World Health Organization. (2014). Ebola and Marburg virus disease epidemics: Preparedness, alert, control, and evaluation.** [**https://www.who.int/publications-detail-redirect/WHO-HSE-PED-CED-2014.05**](https://www.who.int/publications-detail-redirect/WHO-HSE-PED-CED-2014.05)
