# Supplementary figures and images for: Gaps and opportunities for data systems and economics to support priority setting for climate-sensitive infectious diseases in sub-Saharan Africa: A rapid scoping review
Source: PLOS Glob Public Health. 2025 Jun 11;5(6):e0003814. doi: 10.1371/journal.pgph.0003814 (PMC12157337; doi:10.1371/journal.pgph.0003814)

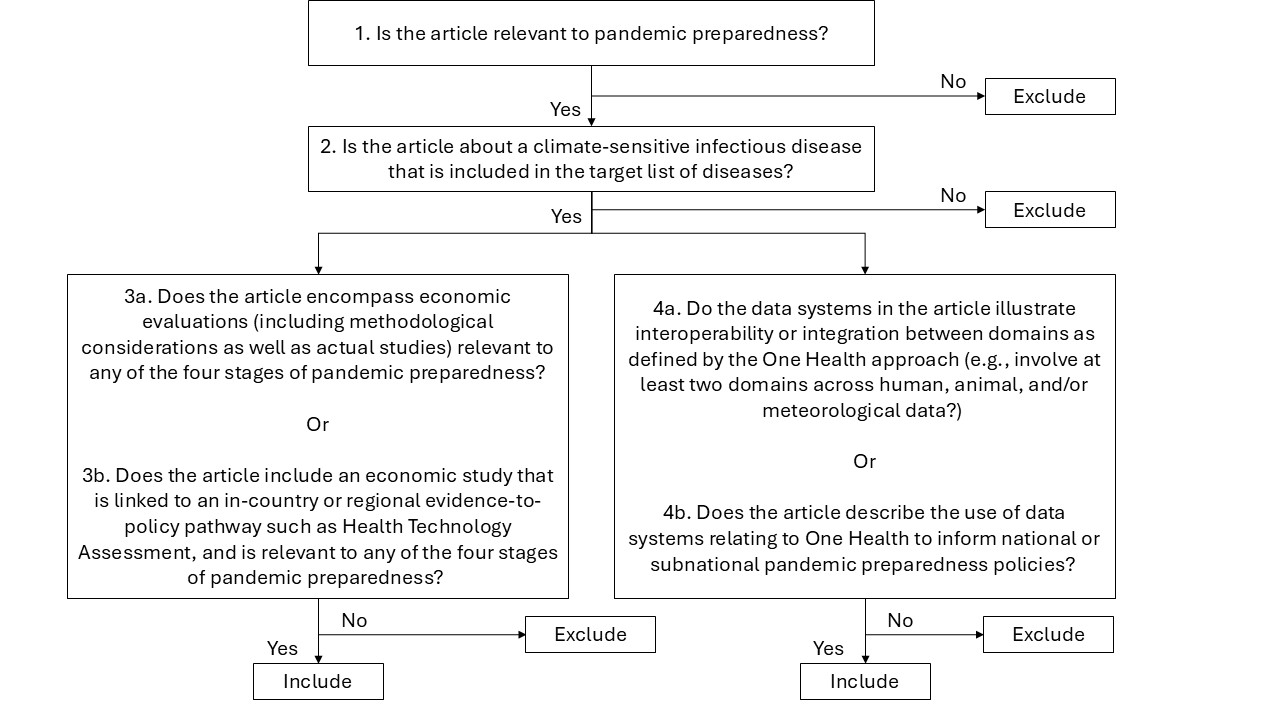


**S2 Fig. Full text screening criteria.**

Supplement: S2 Fig — (DOCX) [file pgph.0003814.s002.docx]
