# Supplementary material for: Gaps and opportunities for data systems and economics to support priority setting for climate-sensitive infectious diseases in sub-Saharan Africa: A rapid scoping review
Source: PLOS Glob Public Health. 2025 Jun 11;5(6):e0003814. doi: 10.1371/journal.pgph.0003814 (PMC12157337; doi:10.1371/journal.pgph.0003814)
Supplement: S3 Fig — Studies could be relevant to all four stages; percentage was calculated as tagged stage by total of tagged stages in data system studies (n = 50) or economic studies (n = 18). (DOCX) [file pgph.0003814.s003.docx]

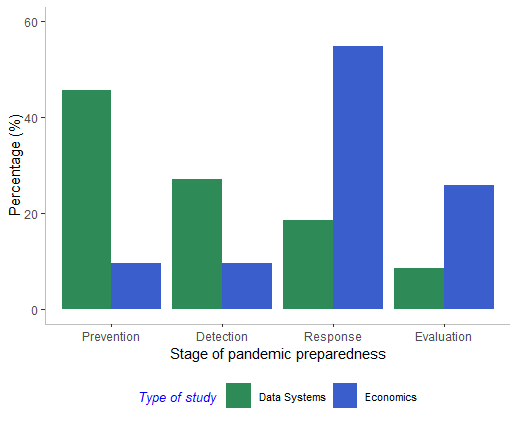


**S3 Fig. Percentage of data system or economic studies mapped to stages of pandemic preparedness. Studies could be relevant to all four stages; percentage was calculated as tagged stage by total of tagged stages in data system studies (n = 50) or economic studies (n = 18).**
