# Supplementary material for: Gaps and opportunities for data systems and economics to support priority setting for climate-sensitive infectious diseases in sub-Saharan Africa: A rapid scoping review
Source: PLOS Glob Public Health. 2025 Jun 11;5(6):e0003814. doi: 10.1371/journal.pgph.0003814 (PMC12157337; doi:10.1371/journal.pgph.0003814)

**S4 Fig. Number of studies published over time, by geographical distribution of author affiliations.**  **The institutional affiliations of authors were categorised as follows: African - all author affiliations located within Africa; African & International - author affiliations located both within Africa and internationally; and International - all author affiliations located outside of Africa.**


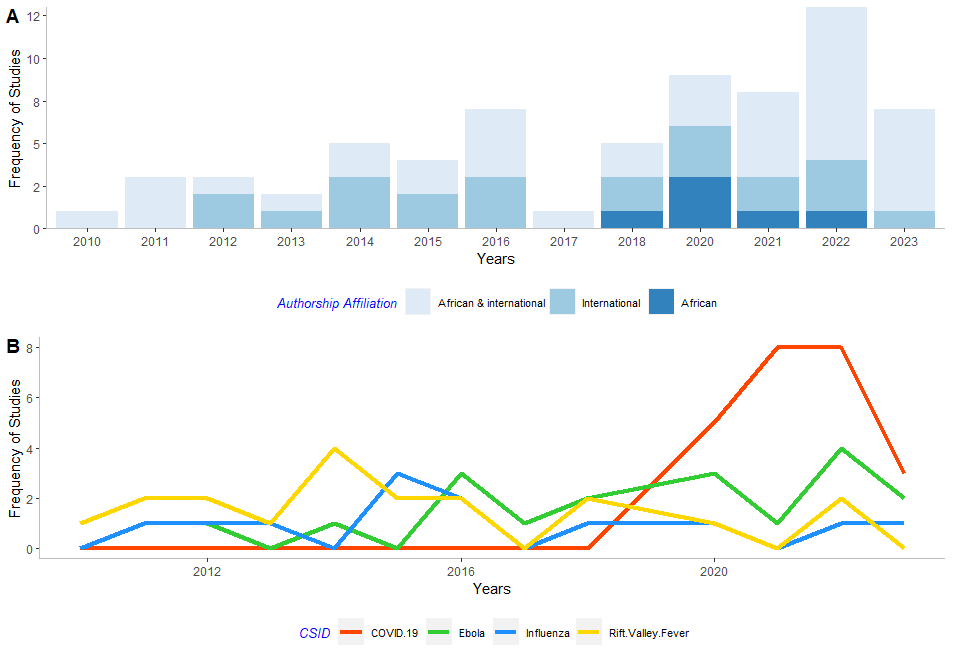

Supplement: S4 Fig — The institutional affiliations of authors were categorised as follows: African - all author affiliations located within Africa; African & International - author affiliations located both within Africa and internationally; and International - all author affiliations located outside of Africa. (DOCX) [file pgph.0003814.s004.docx]
