# Supplementary material for: Gaps and opportunities for data systems and economics to support priority setting for climate-sensitive infectious diseases in sub-Saharan Africa: A rapid scoping review
Source: PLOS Glob Public Health. 2025 Jun 11;5(6):e0003814. doi: 10.1371/journal.pgph.0003814 (PMC12157337; doi:10.1371/journal.pgph.0003814)
Supplement: S1 Table — (DOCX) [file pgph.0003814.s005.docx]

**S1 Table. Screening guidance**

| Question | Framework | Definition(s) | Reference |
| --- | --- | --- | --- |
| *Is the article relevant to pandemic preparedness?* | **Prevention (Stage 1):** Pre-epidemic emergence. Emergence and re-emergence of CSIDs.  **Detection (Stage 2):** Identification and Evaluation of Risk. Surveillance systems, diagnostic testing, risk prediction tools for CSIDs.  **Control (Stage 3):** Outbreak Response and Containment. Efficacy and effectiveness of outbreak control measures.  **Evaluation (Stage 4):** Post-epidemic Evaluation. After-action reviews. | ***Pandemic preparedness*:** the capacity of institutions including public health authorities, health systems and emergency response bodies, to detect, report and respond to outbreaks. | Pandemic Preparedness Framework from:  World Health Organization. (2014). Ebola and Marburg virus disease epidemics: Preparedness, alert, control, and evaluation [1]. |
| *Is the article about a climate-sensitive infectious disease(s) that is included in the target list of diseases?* | **Target CSID pathogen list:**  Influenza A; Ebolavirus and Marburg virus; Zika virus; Severe Acute Respiratory Syndrome; Middle East Respiratory Syndrome; COVID-19; Crimean-Congo Haemorrhagic Fever; Lassa Fever; Nipah and Henipavirus Disease; Rift Valley fever; Mpox; Disease X.    Zoonotic pathogens with a single spill-over event and subsequent human-to-human transmission even if no longer climate-sensitive, are within scope.    Articles about non-target CSIDs will be documented but not included in the final study set. These will be identified through a non-exhaustive searchable reference list of CSIDs (<https://camilo-mora.github.io/Diseases/>). | ***Climate change*:** A change in the state of the climate that can be identified by changes in the mean and/or the variability of its properties and that persists for an extended period, typically decades or longer. Climate change may be due to natural internal processes or external forcings, or to persistent anthropogenic changes in the composition of the atmosphere or in land use.  *Emerging Infectious Disease*: An emerging infectious disease is one that either has appeared in and affected a population for the first time, or has existed previously but is rapidly spreading, either in incidence or geographic range.  ***Zoonotic disease*:** an infectious disease that is naturally transmissible from vertebrate animals to people. | Priority list of pathogens with epidemic potential from:  World Health Organization. (2022). Prioritizing diseases for research and development in emergency contexts [2].  Viral pathogens declared as public health emergencies of international concern (PHEIC) from:  Wilder-Smith, A., & Osman, S. (2020). Public health emergencies of international concern: A historic overview [3]. |
| *Does the article refer to economic evaluation or health economic studies to support priority setting including Health Technology Assessment?* | **Methods to support explicit priority setting:**  Health Technology Assessment  Economic evaluation (cost-effectiveness/utility analysis, cost-benefit analysis, cost-consequence analysis) | ***Economic evaluation*:** the explicit comparison of alternative interventions to support investment decisions that take into account value for money. | Definitions from World Health Organization. (2024). Economic evaluation & Analysis [4]. |
| *Does the article discuss data systems relevant to identified domains for priority setting?* | **Identified domains:**  Publications about or using named (established) data systems, or that describe the creation of new data systems, or that outline the interoperable use of multi-sectoral data systems. | ***Data systems*:** generate information to enable decision-makers at all levels of the health system to identify problems and needs, make evidence-based decisions on health policy and allocate scarce resources optimally. Ideal systems are institutionalised and interoperable to facilitate data sharing across all levels of decision-making.  *Data sets:* collections of information that originate from both primary and secondary sources.  *Data sources:* locations or origins from which data is collected or obtained. Sampling frames are derived from data sources and serve as the basis to creating data sets. | Definitions have been adapted from:  Koon et al. (2020). A scoping review of the uses and institutionalisation of knowledge for health policy in low- and middle-income countries [5]; NHS. (2024). Data Sets [6]; and Mooney & Garber. (2019). Sampling and Sampling Frames in Big Data Epidemiology [7]. |

**References**

1. WHO. Ebola and Marburg virus disease epidemics: preparedness, alert, control, and evaluation. 2014 2014.

2. WHO. Prioritizing diseases for research and development in emergency contexts. Prioritizing diseases for research and development in emergency contexts. 2022.

3. Wilder-Smith A, Osman S. Public health emergencies of international concern: a historic overview. Journal of Travel Medicine. 2020;27(8).

4. World Health O. Economic evaluation & analysis. 2024.

5. Koon AD, Windmeyer L, Bigdeli M, Charles J, El Jardali F, Uneke J, et al. A scoping review of the uses and institutionalisation of knowledge for health policy in low- and middle-income countries. Health Research Policy and Systems. 2020;18(1):7.

6. NHS England. Data Sets [Available from: <https://digital.nhs.uk/data-and-information/data-collections-and-data-sets/data-sets>.

7. Mooney SJ, Garber MD. Sampling and Sampling Frames in Big Data Epidemiology. Curr Epidemiol Rep. 2019;6(1):14-22.
