# Supplementary material for: Gaps and opportunities for data systems and economics to support priority setting for climate-sensitive infectious diseases in sub-Saharan Africa: A rapid scoping review
Source: PLOS Glob Public Health. 2025 Jun 11;5(6):e0003814. doi: 10.1371/journal.pgph.0003814 (PMC12157337; doi:10.1371/journal.pgph.0003814)
Supplement: S2 Table — (DOCX) [file pgph.0003814.s006.docx]

**S2 Table. Summary of Design Data system studies**

| **Author** | **Year of publica-tion** | **Aims** | **CSIDs** | **Data system properties** | | | | **Gender lens** |
| --- | --- | --- | --- | --- | --- | --- | --- | --- |
|  |  |  |  | Name | Scope | Stage of imple-mentation | Description |  |
| Abayomi [1] | 2021 | Describe the efforts of Lagos State in developing preparedness and response plan based on the 2014 EVD outbreak and the deployment of this plan in response to COVID-19. | Ebola; COVID-19 | Incidence Management Centre (later renamed National Emergency Operation Centre) | Local | Operational | Coordinating system used during Lagos state response to EVD and later COVID-19 (and later for national response). | Gender-blind research |
|  |  |  |  | Lagos State Biobank and Biosecurity | National | Operational | Diagnostic laboratory system that stores biological material and associated data. |  |
| Karimuribo [2] | 2012 | Assess the combination of participatory epidemiological approaches and mobile technologies (running EpiCollect) to enhance surveillance and alert in remote areas. | Ebola; Marburg; Rift Valley fever | Community-based Active Surveillance System (part of the Southern Africa One Health surveillance strategy) | Regional | Pilot | CBS system aimed to actively capture disease events in animal and human populations. | Gender-blind research |
|  |  |  |  | EpiCollect | International | Operational | Mobile surveillance tool application for data collection and sharing in near-real time. |  |
|  |  |  |  | District-based Passive Surveillance System (part of the Southern Africa One Health surveillance strategy) | Regional | Pilot | Integrated surveillance system across animal and human health sectors using mobile technologies. |  |
| Liebenberg [3] | 2016 | Demonstrate the value of employing trackers using smartphones in large-scale, long-term monitoring of ecosystems for conservation management. | Ebola | CyberTracker | Regional | Operational | Mobile surveillance tool application for tracking animals to provide near real-time data for use in surveillance and conservation. | Gender-blind research |
| Tourre [4] | 2010 | Investigate the distribution of the *Aedes vexans* density and identify risk zones for the implementation of a new operational early warning system for RVF. | Rift Valley fever | RedGems | International | Operational | EWS using an online interactive tool to predict and mitigate public health impacts from infectious diseases. | Gender-blind research |
| Burke [5] | 2011 | Review the Armed Forces Health Surveillance Center (AFHSC) across zoonosis survey efforts. | Influenza; Crimean-Congo Haemorrhagic Fever; Lassa Fever; Rift Valley fever; Mpox | Joint Biological Agent Identification and Diagnosis System | International | Operational | Laboratory data system that provides real-time polymerase chain reaction analysis of clinical and environmental samples for infectious disease agents. | Gender-blind research |
|  |  |  |  | No name (Integration of Ugandan Wild Bird Surveillance with domestic poultry, swine and human influenza sentinel surveillance) | International | Operational | Integrated surveillance system to detect animal and human outbreaks of influenza. |  |
|  |  |  |  | Integrated Human-Animal-Vector Surveillance | International | Operational | Integrated surveillance to identify variations in circulating influenza strains through follow up of human and animals. |  |
| Tambo [6] | 2018 | Assess the current trends in re-emerging Lassa fever outbreak in understanding spatio-geographical reservoirs, risk factors patterns, and Lassa virus incidence mapping. | Lassa Fever | Community-based "One Health" Surveillance and Emergency Response Practice (not named, suggested system) | National | Proposed | CBS system aimed to address persistent scourge of poverty-related Lassa Fever and other emerging zoonotic disease pandemic threats. | Gender-blind research |
| Guenin [7] | 2022 | Provide preparatory insights for the definition of a community-based surveillance system for emerging zoonoses in Guinea. Additionally explore the disease detection capacity and the surveillance network opportunities at the community level in two pilot areas in the forest region of Guinea. | Ebola; Lassa Fever | Community health information exchange network | Local | Proposed | CBS conceptual community health information exchange network across human, animal and environmental domains based off outputs of focus group discussions. | Gender-specific research |
| Holmes [8] | 2018 | Commentary discussing limitations of predictive genomic technologies and advantages of alternative active surveillance system. | Influenza; Ebola; Severe Acute Respiratory Syndrome (SARS); Middle East respiratory syndrome (MERS) | Global Virome Project | International | Operational | Genomic system based on genomic surveys across mammals and birds to predict potential strains with potential to affect humans. | Gender-blind research |
| Massengo [9] | 2023 | Review studies on arbovirus disease surveillance in Africa utilising a One Health framework, highlighting the importance of integrated strategies and to highlight the issues related to the implementation of a One Health approach in Africa | Influenza; Ebola; COVID-19; Crimean-Congo Haemorrhagic Fever; Rift Valley fever | Suggested integrated surveillance system | Regional | Proposed | Integrated surveillance system aimed at prevention and control of CCHF. | Gender-blind research |
| Hassan [10] | 2014 | Compare two major outbreaks of RVF in Saudi Arabia (2000) and Sudan (2007) from a One Health perspective. | Rift Valley fever | Not named - describes One Health System Surveillance in Sudan. | National | Operational | Integrated surveillance system applied to containing an RVF outbreak in White Nile, Sudan. | Gender-blind research |
| Goutard [11] | 2015 | Review surveillance methods and tools currently developed or implemented by French Research Centre for International Development (CIRAD) researchers in Madagascar and Cambodia. | Influenza; Rift Valley fever | National public and veterinary health services in Madagascar (not named) | National | Operational | Integrated surveillance system based on clinical surveillance on influenza and RVF. | Gender-blind research |
| Zimmerman [12] | 2022 | Review existing great ape surveillance programs in African range habitats to identify successes, gaps, and challenges, and propose the "Great Ape Health Watch" surveillance system that monitors primate health in real-time and generates early warnings of disease outbreaks. | Ebola; Mpox | Animal Mortality Monitoring Network | Regional | Operational | Community-based surveillance system aimed at early detection of outbreaks through community engagement and education, great ape carcass detection and testing, and follow up of surveys for collection of additional epidemiological data. | Gender-blind research |
|  |  |  |  | Great Ape Health Watch using the Spatial Monitoring and Reporting Tool (SMART) | Regional | Proposed | Integrated surveillance system proposed as a global, standardized system that collects, combines and shares indicators of great ape health at different temporal and spatial scales, improving the capacity of national and international actors to detect and respond to outbreaks of disease. |  |
| Baldassi [13] | 2020 | Integrate current prevention and control systems for infectious diseases with a prediction tool (Infectious Disease Seeker (IDS)) able to be used off-line to support decision-makers, health care workers, and first responders to quickly and properly recognise an outbreak. | Influenza; Ebola; Marburg; Zika; Severe Acute Respiratory Syndrome (SARS); Middle East respiratory syndrome (MERS); Lassa Fever; Nipah Virus; Henipah Virus; Rift Valley fever | Infectious Disease Seeker (IDS) | International | Pilot | Outbreak investigation tool able to predict the possible causative agent and related disease, allowing users to compare specific epidemiological parameters (CFR, transmission rate, incubation rate, recovery rate, infectious mortality rate) of two or more diseases, and provides accuracy ratio of estimate. | Gender-sensitive research |
| Oyas [14] | 2018 | Describe the activities and results of enhanced RVF surveillance carried out in Kenya, in response to an early warning alert issued by US Pandemic Prediction and Forecasting Science and Technical Working Group of the National Science and Technology Council, FAO Emergency Prevention Systems, and Kenya Meteorological Department. | Rift Valley fever | Not named, referred to as "enhanced RVF surveillance system" | National | Pilot | EWS based on a piloted system created in response to RVF warning. Aimed at the collection of near real-time data on syndromes and risk factors associated with RVF to enhance early detection of the disease in livestock before spill over to humans. | Gender-blind research |
|  |  |  |  | Kenya Animal Bio surveillance System (KABS) | National | Pilot | EWS based on the syndromic surveillance of domestic and wild animals to detect and report animal health status and assist decision-making and response. Currently being developed by US CDC in response to the piloted RVF enhanced surveillance system. |  |
| Zhao [15] | 2022 | The study formulated indicators for zoonoses and applied OH principles (capacity of a country to respond/ prevent zoonotic events associated with holistic health of the human-animal-environment interface) to data retrieved from publicly available repositories to systematically analyse the OH index for zoonoses in sub-Saharan Africa, and apply to five zoonoses case studies (only COVID-19 as target CSID) | COVID-19 | One Health Zoonosis Index (OHIZ) database and Global One Health Indicator (GOHI) algorithm | Regional | Operational | Capacity system used by authors to incorporate zoonoses datasets from different sources and apply a One Health Zoonosis Index (OHIZ) to score sub-Saharan African countries capacity to cope with zoonoses across surveillance, vector control, sanitation, policy, and more. | Gender-blind research |
| Ramadan [16] | 2022 | Understand the epidemiology of RVF outbreak that occurred in South Sudan 2017-2018; to examine the country's laboratory capacity; to identify barriers to application of the One Health approach in investigating zoonosis; and to develop recommendations to strengthen One Health approach in South Sudan. | Rift Valley fever | Early Warning and Response Network (EWARN) | National | Operational | EWS able to capture infectious diseases, including those with pandemic potential. | Gender-sensitive research |
| Witt [17] | 2011 | Report on The Armed Forces Health Surveillance Center, Division of Global Emerging Infections Surveillance and Response System Operations (AFHSC-GEIS) predictive surveillance program, who's ultimate goal is pro-active public health practice through pre-event preparedness, prevention and control, and response decision-making and prioritization. | Ebola; Crimean-Congo Haemorrhagic Fever; Rift Valley fever | AFHSC-GEIS predictive surveillance program. | International | Operational | EWS aimed at providing the department of defence decision-makers with advanced awareness on emerging infectious disease threats, and thereby promote timely, science- based disease outbreak prevention, preparedness, and control-and-response action. | Gender-blind research |
|  |  |  |  | MosquitoMap | International | Operational | Mapping communication tool organises and standardises mosquito collection, distribution data, and pathogen-transmission models, paired with a raster overlay analytic tool to map and quantifies extent of where vectors, disease pathogens, and humans co-occur. |  |
| Meseko* [18] | 2015 | Investigate the introduction and spread of pandemic H1N1 virus in humans and animals in Africa by analysing data on the molecular and phylogenetic features. | Influenza | National Center for Biological Information (NCBI) Influenza resources, GISAID, GenBank | International | Operational | Genomic system that stores gene sequences of novel A/H1N1 viruses. | Gender-blind research |
| Lafaye* [19] | 2013 | Report on activities of the “Adaptation à la Fièvre de la Vallée du Rift” (AdaptFVR) project: (i) to produce - in near real-time - validated risk maps for parked live-stock exposed to RVF mosquitoes/vectors bites; (ii) to assess the impacts on RVF vectors from climate variability at different time-scales including climate change; and (iii) to isolate processes improving local livestock management and animal health. | Rift Valley fever | RVFews | National | Pilot | EWS against Rift Valley fever based on sharing risk with stakeholders, the system was used to share eight weekly bulletins with dynamic maps (illustrating zones potentially occupied by mosquitoes) and proper interpretation to relevant stakeholders during the 2010 rainy season, from 7 July to 1 October 2010. | Gender-blind research |
| Pigott* [20] | 2016 | Update a previous ecological niche study (Pigott et al., 2014) with new Ebola occurrence and species data and disseminate results via ViZHub tool. | Ebola | VizHub | International | Operational | Mapping communication tool that allows users to interrogate ecological niche maps in more detail and in areas of specific interest. | Gender-blind research |

*Categorised as both Design and Usage data system studies. Abbreviations: EWS: Early warning system

**References**

1. Abayomi A, Balogun MR, Bankole M, Banke-Thomas A, Mutiu B, Olawepo J, et al. From Ebola to COVID-19: emergency preparedness and response plans and actions in Lagos, Nigeria. Global Health. 2021;17(1):79.

2. Karimuribo ED, Sayalel K, Beda E, Short N, Wambura P, Mboera LG, et al. Towards one health disease surveillance: the Southern African Centre for Infectious Disease Surveillance approach. The Onderstepoort journal of veterinary research. 2012;79(2):454.

3. Liebenberg L, Steventon J, Brahman I, Benadie K, Minye J, Langwane H, et al. Smartphone Icon User Interface design for non-literate trackers and its implications for an inclusive citizen science. Biological Conservation. 2017;208:155-62.

4. Tourre YM, Lacaux JP, Vignolles C, Ndione JA, Lafaye M, editors. Rift Valley Fever (RVF) risks in Senegal using high-resolution remote sensing2010; Kyoto, JAPAN.

5. Burke RL, Kronmann KC, Daniels CC, Meyers M, Byarugaba DK, Dueger E, et al. A review of zoonotic disease surveillance supported by the Armed Forces Health Surveillance Center. Zoonoses and public health. 2012;59(3):164-75.

6. Tambo E, Adetunde OT, Olalubi OA. Re-emerging Lassa fever outbreaks in Nigeria: Re-enforcing "One Health" community surveillance and emergency response practice. Infectious diseases of poverty. 2018;7(1):37.

7. Guenin M-J, De Nys HM, Peyre M, Loire E, Thongyuan S, Diallo A, et al. A participatory epidemiological and One Health approach to explore the community's capacity to detect emerging zoonoses and surveillance network opportunities in the forest region of Guinea. PLoS neglected tropical diseases. 2022;16(7):e0010462.

8. Holmes EC, Rambaut A, Andersen KG. Pandemics: Spend on surveillance, not prediction comment. Nature. 2018;558(7709):180-2.

9. Massengo NRB, Tinto B, Simonin Y. One health approach to arbovirus control in Africa: interests, challenges, and difficulties. Microorganisms. 2023;11(6).

10. Hassan OA, Ahlm C, Evander M. A need for one health approach-lessons learned from outbreaks of Rift Valley fever in Saudi Arabia and Sudan. Infection Ecology and Epidemiology. 2014;4(1):20710.

11. Goutard FL, Binot A, Duboz R, Rasamoelina-Andriamanivo H, Pedrono M, Holl D, et al. How to reach the poor? Surveillance in low-income countries, lessons from experiences in Cambodia and Madagascar. Preventive Veterinary Medicine. 2015;120(1):12-26.

12. Zimmerman DM, Mitchell SL, Wolf TM, Deere JR, Noheri JB, Takahashi E, et al. Great ape health watch: enhancing surveillance for emerging infectious diseases in great apes. Special Issue: One health. 2022;84(4/5).

13. Baldassi F, Cenciarelli O, Malizia A, Gaudio P. First Prototype of the Infectious Diseases Seeker (IDS) Software for Prompt Identification of Infectious Diseases. Journal of epidemiology and global health. 2020;10(4):367-77.

14. Oyas H, Holmstrom L, Kemunto NP, Muturi M, Mwatondo A, Osoro E, et al. Enhanced surveillance for Rift Valley Fever in livestock during El Nino rains and threat of RVF outbreak, Kenya, 2015-2016. PLoS neglected tropical diseases. 2018;12(4):e0006353.

15. Zhao HQ, Fei SW, Yin JX, Li Q, Jiang TG, Guo ZY, et al. Assessment of performance for a key indicator of One Health: evidence based on One Health index for zoonoses in Sub-Saharan Africa. Infectious Diseases of Poverty. 2022;11(1):109.

16. Ramadan OPC, Berta KK, Wamala JF, Maleghemi S, Rumunu J, Ryan C, et al. Analysis of the 2017-2018 Rift valley fever outbreak in Yirol East County, South Sudan: a one health perspective. The Pan African medical journal. 2022;42(Suppl 1):5.

17. Witt CJ, Richards AL, Masuoka PM, Foley DH, Buczak AL, Musila LA, et al. The AFHSC-Division of GEIS Operations Predictive Surveillance Program: a multidisciplinary approach for the early detection and response to disease outbreaks. BMC public health. 2011;11 Suppl 2:S10.

18. Meseko CA, Odurinde OO, Olaniran BO, Heidari A, Oluwayelu DO. Pandemic influenza A/H1N1 virus incursion into Africa: countries, hosts and phylogenetic analysis. Nigerian Veterinary Journal. 2015;36(3):1251-61.

19. Lafaye M, Sall B, Ndiaye Y, Vignolles C, Tourre YM, Borchi FO, et al. Rift Valley fever dynamics in Senegal: a project for pro-active adaptation and improvement of livestock raising management. Geospatial health. 2013;8(1):279-88.

20. Pigott DM, Millear AI, Earl L, Morozoff C, Han BA, Shearer FM, et al. Updates to the zoonotic niche map of Ebola virus disease in Africa. eLife. 2016;5.
