# Supplementary material for: Gaps and opportunities for data systems and economics to support priority setting for climate-sensitive infectious diseases in sub-Saharan Africa: A rapid scoping review
Source: PLOS Glob Public Health. 2025 Jun 11;5(6):e0003814. doi: 10.1371/journal.pgph.0003814 (PMC12157337; doi:10.1371/journal.pgph.0003814)
Supplement: S3 Table — (DOCX) [file pgph.0003814.s007.docx]

**S3 Table: Summary of Usage Data System studies**

| **Author** | **Year of publication** | **Aims** | **CSIDs** | **Type of study** | **Data domains integrated** | **Gender lens** |
| --- | --- | --- | --- | --- | --- | --- |
| Aidoo [1] | 2021 | Contribute to knowledge on the effect of weather on the spread of COVID-19 infection in Ghana. | COVID-19 | Association | Human; Environmental | Gender-blind research |
| Anyamba [2] | 2014 | Illustrate the impacts of weather extremes on RVF transmission, agriculture, and vector-borne disease outbreak patterns around the world. | Rift Valley fever | Association | Animal; Environmental | Gender-blind research |
| Anyamba [3] | 2022 | Understand the effects of climate and weather on RVF mosquito vector populations. | Rift Valley fever | Association | Animal; Environmental | Gender-blind research |
| Azziz-Baumgartner [4] | 2012 | Compare the epidemic influenza activity in tropical, subtropical, and temperate countries. | Influenza | Association | Human; Environmental | Gender-blind research |
| Boufekane [5] | 2021 | Investigate the correlation among COVID-19 spread and local environmental factors within fourteen cities of Algeria. | COVID-19 | Association | Human; Environmental | Gender-blind research |
| Cambaza [6] | 2020 | Analyse the relationships between weather (temperature and atmospheric pressure) and the frequency of confirmed COVID-19 cases in Mozambique. | COVID-19 | Association | Human; Environmental | Gender-blind research |
| Diouf [7] | 2021 | Investigate the potential contribution of climate conditions on COVID-19 pandemic transmission in 16 highly populated West and North African countries divided into three climatic regions (Maghreb, Sahel, and the Gulf of Guinea) | COVID-19 | Association | Human; Environmental | Gender-blind research |
| McKendrick [8] | 2023 | Apply a periodically forced seasonal nonautonomous system of ordinary differential equations as a vector model to demonstrate that the population dynamics of the Lassa Fever rodent reservoir may be responsible for the spikes in the number of observed Lassa Fever cases in humans. | Lassa Fever | Association | Human; Animal | Gender-blind research |
| Meo [9] | 2020 | Investigate the impact of heat and humidity on daily basis incidence and mortality due to COVID-19 pandemic in ten of the world's hottest countries compared to ten of the coldest ones. | COVID-19 | Association | Human; Environmental | Gender-blind research |
| Pana [10] | 2021 | Identify the country-level determinants of the severity of the first wave of the COVID-19 pandemic. | COVID-19 | Association | Human; Environmental | Gender-blind research |
| Arruda [11] | 2023 | Identify surveillance gaps and quantify some of the current biases in genomic surveillance, by performing a study of LASV nucleotide sequences available from the National Centre for Biotechnology Information GenBank using associated metadata to spatially model sequencing effort. | Lassa Fever | Phylogenetic | Human; Animal | Gender-blind research |
| Chan [12] | 2013 | Propose a readily interpretable and computable quantitative measurement for genomic surveillance of a pathogen that directly accounts for the number of isolates, the evolutionary rate, and the time of sample collection without the need to define arbitrary clades or species or the need for a full phylogenetic reconstruction. | Influenza | Phylogenetic | Human; Animal | Gender-blind research |
| Olayemi [13] | 2020 | Phylogenetically infer ancestry and descent between LASV sequences detected in rodents and humans in selected localities within West Africa to provide increased insight into virus transmission at the rodent-human boundary, and compile a dataset including LASV sequences collected from both humans and Mastomys rodents. | Lassa Fever | Phylogenetic | Human; Animal | Gender-blind research |
| Arsevska [14] | 2014 | Determine geographic and temporal variations in risk of RVF in the Maghreb, using multicriteria decision analysis (MCDA) to map hotspots and time periods of high-risk, and produce vector distribution maps. | Rift Valley fever | Risk mapping | Animal; Environmental | Gender-blind research |
| Bogoch [15] | 2016 | Identify regions and times where the potential health, economic, and social effects from Zika virus are greatest, focusing on resource-limited countries in Africa and the Asia-Pacific region. | Zika | Risk mapping | Human; Animal; Environmental | Gender-blind research |
| Brown [16] | 2015 | Demonstrate two methods for constructing a bio surveillance network based on a risk map and validate the county risk map with data from a known outbreak in Nigeria. | Influenza | Risk mapping | Human; Animal | Gender-blind research |
| Guilloteau [17] | 2014 | Investigate the use of high-resolution satellite rainfall products and see if the prediction of pond dynamics and the RVF vector risk assessment can be improved. | Rift Valley fever | Risk mapping | Animal; Environmental | Gender-blind research |
| Jagadesh [18] | 2020 | Measure predictive risks of CSIDs from the WHO list of R&D blueprint pathogens, and evaluate the use of biogeography on predicting diseases outbreaks, including identifying potential hotspots for Disease X. | Ebola; Marburg; COVID-19; Nipah Virus; Rift Valley fever; Disease X | Risk mapping | Human; Animal; Environmental | Gender-blind research |
| Nyakarahuka [19] | 2017 | Predict where filovirus outbreaks are likely to occur in Uganda to guide epidemic preparedness and surveillance. | Ebola; Marburg | Risk mapping | Human; Animal; Environmental | Gender-blind research |
| Ochieng [20] | 2016 | Evaluate the effect of climate change on RVF vector distribution in Baringo County, Kenya, with an aim of developing a risk map for spatial prediction of RVF outbreaks. | Rift Valley fever | Risk mapping | Animal; Environmental | Gender-blind research |
| Pigott [21] | 2014 | Define the areas suitable for zoonotic transmission of Ebolavirus through species distribution modelling techniques. | Ebola | Risk mapping | Human; Animal | Gender-blind research |
| Simons [22] | 2023 | Synthesise data from West Africa from 127 rodent trapping studies as an additional source of information to characterise the range and presence of rodent species and identify the sub-group of species that are potential or known pathogen hosts. | Lassa Fever | Risk mapping | Human; Animal; Environmental | Gender-blind research |
| Mahama [23] | 2022 | Assess the epidemiological transition, pathogen-host interactions, spatiotemporal distribution, transmission routes, and their potential areas of impact to understand the risk and epidemiological landscape of the spectrum of IDs in Ghana as a case study for SSA. | Influenza; COVID-19 | Risk mapping | Human; Animal | Gender-blind research |
| Ameh Yaro [24] | 2020 | Evaluate the impact of selected demographic and environmental variables to identify potential risk areas and hotspots for SARS-CoV-2 transmission in Nigeria. | COVID-19 | Risk mapping & association | Human; Environmental | Gender-blind research |
| Anyamba [25] | 2012 | Analyse a variety of climate and satellite-derived vegetation measurements to explain the coupling between patterns of climate variability and disease outbreaks of Rift Valley fever and chikungunya. | Rift Valley fever | Risk mapping & association | Human; Animal; Environmental | Gender-blind research |
| Fuller [26] | 2011 | Use remote sensing and epidemiological surveillance data to identify ecological risk factors for contracting human monkeypox. | Mpox | Risk mapping & association | Human; Animal; Environmental | Gender-blind research |
| Glancey [27] | 2015 | Investigate the association of Normalized Difference Vegetation Index data, as a surrogate for rainfall, soil moisture, soil type, and energy availability with RVF outbreaks over 2008-2011. | Rift Valley fever | Risk mapping & association | Human; Animal | Gender-blind research |
| Lo Iacono [28] | 2018 | Apply a unified, process-based model built on realistic representation of how water body dynamics obtained from satellite images and temperature influence the ecology of the primary mosquito vectors and epidemiology of RVF. | Rift Valley fever | Risk mapping & association | Animal; Environmental | Gender-blind research |
| Mosomtai [29] | 2016 | Identify ecological factors that explain the risk of RVF outbreaks in eastern and central Kenya and produce a spatially explicit risk map. | Rift Valley fever | Risk mapping & association | Animal; Environmental | Gender-blind research |
| Walsh [30] | 2016 | (i) Delineate areas at greatest risk of H5N1 epizootics among domestic poultry, (ii) identify abiotic and biotic features of the landscape associated with outbreak risk and (iii) examine patterns of epizootic clustering by season. | Influenza | Risk mapping & association | Animal; Environmental | Gender-blind research |
| Meseko* [31] | 2015 | Investigate the introduction and spread of pandemic H1N1 virus in humans and animals in Africa by analysing data on the molecular and phylogenetic features. | Influenza | Phylogenetic | Human; Animal | Gender-blind research |
| Lafaye* [32] | 2013 | Report on activities of the “Adaptation à la Fièvre de la Vallée du Rift” (AdaptFVR) project: (i) to produce - in near real-time - validated risk maps for parked live-stock exposed to RVF mosquitoes/vectors bites; (ii) to assess the impacts on RVF vectors from climate variability at different time-scales including climate change; and (iii) to isolate processes improving local livestock management and animal health. | Rift Valley fever | Risk mapping | Animal; Environmental | Gender-blind research |
| Pigott* [33] | 2016 | Update a previous ecological niche study (Pigott et al., 2014) with new Ebola occurrence and species data and disseminate results via ViZHub tool. | Ebola | Risk mapping | Human; Animal; Environmental | Gender-blind research |

*Categorised as both Design and Usage data system studies.

**References**

1. Aidoo EN, Adebanji AO, Awashie GE, Appiah SK. The effects of weather on the spread of COVID-19: evidence from Ghana. Bulletin of the National Research Centre. 2021;45(1):20.

2. Anyamba A, Small JL, Britch SC, Tucker CJ, Pak EW, Reynolds CA, et al. Recent weather extremes and impacts on agricultural production and vector-borne disease outbreak patterns. PloS one. 2014;9(3):e92538.

3. Anyamba A, Damoah R, Kemp A, Small JL, Rostal MK, Bagge W, et al. Climate Conditions During a Rift Valley Fever Post-epizootic Period in Free State, South Africa, 2014-2019. Frontiers in veterinary science. 2021;8:730424.

4. Azziz-Baumgartner E, Dao C, Nasreen S, Bhuiyan MU, Munir ME, Al Mamun A, et al. Seasonality, timing, and climate drivers of influenza activity worldwide. American Journal of Tropical Medicine and Hygiene. 2011;85(6 SUPPL. 1):427.

5. Boufekane A, Busico G, Maizi D. Effects of temperature and relative humidity on the COVID-19 pandemic in different climates: a study across some regions in Algeria (North Africa). Environmental Science and Pollution Research. 2021;29(12):18077-102.

6. Cambaza EM, Viegas GC, Cambaza CM. Potential impact of temperature and atmospheric pressure on the number of cases of COVID-19 in Mozambique, Southern Africa. Journal of Public Health and Epidemiology. 2020;12(3):246-60.

7. Diouf I, Sy S, Senghor H, Fall P, Diouf D, Diakhate M, et al. Potential Contribution of Climate Conditions on COVID-19 Pandemic Transmission over West and North African Countries. Atmosphere. 2022;13(1).

8. McKendrick JQ, Tennant WSD, Tildesley MJ. Modelling seasonality of Lassa fever incidences and vector dynamics in Nigeria. PLoS neglected tropical diseases. 2023;17(11):e0011543.

9. Meo SA, Abukhalaf AA, Alomar AA, Al-Beeshi IZ, Alhowikan A, Shafi KM, et al. Climate and COVID-19 pandemic: effect of heat and humidity on the incidence and mortality in world's top ten hottest and top ten coldest countries. European review for medical and pharmacological sciences. 2020;24(15):8232-8.

10. Pana TA, Bhattacharya S, Gamble DT, Pasdar Z, Szlachetka WA, Perdomo-Lampignano JA, et al. Country-level determinants of the severity of the first global wave of the COVID-19 pandemic: an ecological study. BMJ open. 2021;11(2):e042034.

11. Arruda LB, Free HB, Simons D, Ansumana R, Elton L, Haider N, et al. Current sampling and sequencing biases of Lassa mammarenavirus limit inference from phylogeography and molecular epidemiology in Lassa fever endemic regions. PLOS global public health. 2023;3(11):e0002159.

12. Chan JM, Rabadan R. Quantifying pathogen surveillance using temporal genomic data. mBio. 2013;4(1):e00524-12.

13. Olayemi A, Adesina AS, Strecker T, Magassouba NF, Fichet-Calvet E. Determining Ancestry between Rodent- and Human-Derived Virus Sequences in Endemic Foci: Towards a More Integral Molecular Epidemiology of Lassa Fever within West Africa. Biology. 2020;9(2).

14. Arsevska E, Hellal J, Mejri S, Hammami S, Marianneau P, Calavas D, et al. Identifying Areas Suitable for the Occurrence of Rift Valley Fever in North Africa: Implications for Surveillance. Transboundary and emerging diseases. 2016;63(6):658-74.

15. Bogoch II, Brady OJ, Kraemer MUG, German M, Creatore MI, Brent S, et al. Potential for Zika virus introduction and transmission in resource-limited countries in Africa and the Asia-Pacific region: a modelling study. The Lancet Infectious Diseases. 2016;16(11):1237-45.

16. Brown M, Moore L, McMahon B, Powell D, Labute M, Hyman JM, et al. Constructing Rigorous and Broad Biosurveillance Networks for Detecting Emerging Zoonotic Outbreaks. PLOS ONE. 2015;10(5):e0124037.

17. Guilloteau C, Gosset M, Vignolles C, Alcoba M, Tourre YM, Lacaux J-P. Impacts of Satellite-Based Rainfall Products on Predicting Spatial Patterns of Rift Valley Fever Vectors*. Journal of Hydrometeorology. 2014;15(4):1624-35.

18. Jagadesh S, Combe M, Nacher M, Gozlan R. In search for the hotspots of Disease X: A biogeographic approach to mapping the predictive risk of WHO's blueprint priority diseases. International Journal of Infectious Diseases. 2020;101(Supplement 1):220.

19. Nyakarahuka L, Ayebare S, Mosomtai G, Kankya C, Lutwama J, Mwiine FN, et al. Ecological Niche Modeling for Filoviruses: A Risk Map for Ebola and Marburg Virus Disease Outbreaks in Uganda. PLoS currents. 2017;9.

20. Ochieng AO, Nanyingi M, Kipruto E, Ondiba IM, Amimo FA, Oludhe C, et al. Ecological niche modelling of Rift Valley fever virus vectors in Baringo, Kenya. Infection ecology & epidemiology. 2016;6:32322.

21. Pigott D, Golding N, Mylne A, Huang Z, Henry A, Weiss D, et al. Mapping the Zoonotic niche of Ebola virus disease in Africa. American Journal of Tropical Medicine and Hygiene. 2014;91(5 SUPPL. 1):590.

22. Simons D, Attfield LA, Jones KE, Watson-Jones D, Kock R. Rodent trapping studies as an overlooked information source for understanding endemic and novel zoonotic spillover. PLoS Neglected Tropical Diseases. 2023;17(1):e0010772.

23. Mahama PNJ, Kabo-Bah AT, Blanford JI, Yamba EI, Antwi-Agyei P. Reviewing the Past, Present, and Future Risks of Pathogens in Ghana and What This Means for Rethinking Infectious Disease Surveillance for Sub-Saharan Africa. Journal of Tropical Medicine. 2022;2022:4589007.

24. Ameh Yaro C, Udama Eneche PS, Abu Anyebe D. Risk analysis and hot spots detection of SARS-CoV-2 in Nigeria using demographic and environmental variables: an early assessment of transmission dynamics. International journal of environmental health research. 2022;32(5):1111-22.

25. Anyamba A, Linthicum KJ, Small JL, Collins KM, Tucker CJ, Pak EW, et al. Climate teleconnections and recent patterns of human and animal disease outbreaks. PLoS neglected tropical diseases. 2012;6(1):e1465.

26. Fuller T, Thomassen HA, Mulembakani PM, Johnston SC, Lloyd-Smith JO, Kisalu NK, et al. Using remote sensing to map the risk of human monkeypox virus in the Congo Basin. EcoHealth. 2011;8(1):14-25.

27. Glancey MM, Anyamba A, Linthicum KJ. Epidemiologic and Environmental Risk Factors of Rift Valley Fever in Southern Africa from 2008 to 2011. Vector borne and zoonotic diseases (Larchmont, NY). 2015;15(8):502-11.

28. Lo Iacono G, Cunningham AA, Bett B, Grace D, Redding DW, Wood JLN. Environmental limits of Rift Valley fever revealed using ecoepidemiological mechanistic models. Proceedings of the National Academy of Sciences of the United States of America. 2018;115(31):E7448-E56.

29. Mosomtai G, Evander M, Sandstrom P, Ahlm C, Sang R, Hassan OA, et al. Association of ecological factors with Rift Valley fever occurrence and mapping of risk zones in Kenya. International journal of infectious diseases : IJID : official publication of the International Society for Infectious Diseases. 2016;46:49-55.

30. Walsh MG, Amstislavski P, Greene A, Haseeb MA. The landscape epidemiology of seasonal clustering of highly pathogenic avian influenza (H5N1) in domestic poultry in Africa, Europe and Asia. Transboundary and Emerging Diseases. 2017;64(5):1465-78.

31. Meseko CA, Odurinde OO, Olaniran BO, Heidari A, Oluwayelu DO. Pandemic influenza A/H1N1 virus incursion into Africa: countries, hosts and phylogenetic analysis. Nigerian Veterinary Journal. 2015;36(3):1251-61.

32. Lafaye M, Sall B, Ndiaye Y, Vignolles C, Tourre YM, Borchi FO, et al. Rift Valley fever dynamics in Senegal: a project for pro-active adaptation and improvement of livestock raising management. Geospatial health. 2013;8(1):279-88.

33. Pigott DM, Millear AI, Earl L, Morozoff C, Han BA, Shearer FM, et al. Updates to the zoonotic niche map of Ebola virus disease in Africa. eLife. 2016;5.
