# Supplementary material for: Gaps and opportunities for data systems and economics to support priority setting for climate-sensitive infectious diseases in sub-Saharan Africa: A rapid scoping review
Source: PLOS Glob Public Health. 2025 Jun 11;5(6):e0003814. doi: 10.1371/journal.pgph.0003814 (PMC12157337; doi:10.1371/journal.pgph.0003814)
Supplement: Attachment — Submitted filename: Response to reviewer comments_CSID_PP_v2.docx [file pgph.0003814.s009.docx]

# Individual responses to journal and reviewer comments

This document provides responses to individual journal and reviewer comments. Please note that the line references refer to the Marked Up Revised Manuscript which details the tracked changes.

## **Journal comments**

1. Figure 3: please (a) provide a direct link to the base layer of the map (i.e., the country or region border shape) and ensure this is also included in the figure legend; and (b) provide a link to the terms of use / license information for the base layer image or shapefile. We cannot publish proprietary or copyrighted maps (e.g. Google Maps, Mapquest) and the terms of use for your map base layer must be compatible with our CC-BY 4.0 license.

- *Response*
- *(a) Direct Link to the Base Layer:
  The base layer of the map, showing country and region borders, was sourced from Natural Earth, a public domain geographic data source. The specific shapefiles were accessed using the rnaturalearth package in R. A direct link to the base layer data is provided here:* [*https://www.naturalearthdata.com/downloads/*](https://www.naturalearthdata.com/downloads/)*. This has been included in the figure legend from line 389.*
- ***(****b) Terms of Use:
  Natural Earth data is in the public domain and can be freely used, modified, and redistributed. The terms of use for the base layer are available here:* [*https://www.naturalearthdata.com/about/terms-of-use/*](https://www.naturalearthdata.com/about/terms-of-use/)*. These terms are compatible with the CC-BY 4.0 license.*

## **Reviewer comments**

### Reviewer #1:

**Major comments:**

1. Given that the first objective is to “map key themes and entry points for future African-led research,” it would be helpful to have a positionality statement (or equivalent). If the lead author(s) are not African, some reflection on this is needed.

- *Response*
- *We agree and have added a positionality statement under acknowledgements to the manuscript from line 833: This positionality statement acknowledges that the study focuses on climate-sensitive infectious disease studies in Africa, with a non-African lead author. While the lead author is a British national affiliated with a British institution, the research team includes researchers from and based in The Gambia, Kenya, and Nigeria. To ensure methodological rigor, the review was conducted following established PRISMA-ScR guidelines, minimising the influence of individual biases on results. The authors acknowledge that interpretations in the discussion can be shaped by training, institutional affiliations, and lived experiences. However, the diverse and heterogeneous research team collaboratively contributed to the analysis and interpretations, enriching the discussion with multiple perspectives. The study also recognises the broader issue of limited studies led by senior African authors. By foregrounding the voices and perspectives of African researchers, this review seeks to ensure their contributions are understood within their local and regional contexts, advancing a more inclusive and representative understanding of climate-sensitive infectious disease research in Africa.*

2. This is an extremely ambitious project that deals with a timely and high priority issue. However, I’m left feeling like it’s trying to do “too much.” The narrative review would be more than sufficient as a standalone paper, so adding in the bibliometric analysis and the thematic mapping (and even possibly the gender analysis, which didn’t seem truly intersectional to me) (a) makes it a bit difficult to grasp the study’s key objectives, and (b) gives insufficient attention to these important elements. I would suggest significantly narrowing the scope of this review, or at minimum move some content to appendices.

- *Response*
- *We thank the reviewer for their constructive feedback regarding the scope of our project. We appreciate the issue raised on the challenges of conveying our key objectives amidst the comprehensive analyses presented. We believe that integrating the narrative review, bibliometric analysis, thematic mapping, and gender analysis is important and necessary to comprehensively identify current gaps and opportunities in the field of pandemic preparedness. For example, the bibliometric analysis highlights the breadth of data systems studies across the WHO region and across CSIDs, however the narrative synthesis reveals the heterogeneity in study types and lack of definitions or guidelines in data system literature. Therefore, we believe that these different analyses complement each other and together provide greater evidence than any of them could offer separately.*

*In order to address this comment and streamline the manuscript, we have made the following changes:*

- *The pandemic preparedness topic mapping result has been moved to the supplementary information*
- *Supporting text is provided in the methods section from line 289: “Topic mapping was conducted by categorising the proportion of data system or economic studies relevant to each CSID. Studies were also mapped to each stage of pandemic preparedness, with these results provided in the supplementary information (S3 Fig)”.*
- *The narrative synthesis has been reduced and streamlined; specifically, we replaced a long paragraph with a concise summary at line 456: “The varying scopes of these data systems highlight the breadth of data sharing for CSID outbreak detection and response, spanning local (36, 46), national (32, 38, 40, 43, 46, 47, 49), regional (33, 34, 39, 41, 44), and international levels (30, 31, 33, 35, 37, 42, 45, 48).*
- *The gender analysis has been refocused to discuss gender exclusively, removing the term “intersectionality” from various places, without reference to other social determinants of health. This revision also clarifies the focus of the gender analysis by labelling it as the application of a gender lens rather than an intersectional gender analysis, for example in the methods the analysis is described from line 310* “*A gender lens was applied to studies, by assessing the extent that gender was considered across the studies against definitions provided by the WHO intersectionality gender toolkit for research on infectious diseases of poverty”*

3. Some more justification on why COVID-19 was included as a CSID is needed. I understand initial spillover was (almost certainly) zoonotic, but I’m assuming all of the literature you reviewed dealt with subsequent person-to-person transmission, which is not really climate-sensitive.

- *Response*
- *We included COVID-19 based on its characterisation as such on the database of evidence on climate-sensitive diseases (*[*Mora et al., 2022*](https://pubmed.ncbi.nlm.nih.gov/35968032/)*). Notably, we did not limit inclusion of CSIDs based on if they were animal-to-human transmission only, but also included person-to-person, as climatic patterns can affect social mixing, mobility patterns, and respiratory health. From the database of CSIDs, Mora et al. characterised COVID-19 as a CSID due to evidence linking changes in precipitation and temperature affecting human social gatherings and subsequent increase in human-human transmission, and how warming was associated with reduced spread of COVID-19.* [*Ford et al., 2022*](https://www.thelancet.com/journals/lanplh/article/PIIS2542-5196(22)00174-7/fulltext) *in their Personal View, explain how climatic risks affect transmission of COVID-19, describing that temperature, wind, and humidity influence COVID-19 transmission in ways not fully understood, despite non-climatic factors appearing more important in explaining disease transmission. Therefore we believe COVID-19 is justified to be included in this analysis.*
- *We have clarified our inclusion of COVID-19 as a CSID (Table 1): “Articles referring to CSIDs with pandemic potential. Infectious diseases with pandemic potential were derived from WHO R&D Blueprint Disease List (7) and pathogens declared in PHEIC disease outbreaks (5). These were compared with a database of CSIDs to confirm their climate sensitivity (1). Climate sensitivity is based on published associations with climate change variables, including weather, hydrometeorological hazards, and land-use changes over short or long (decadal) timescales. Zoonotic CSIDs that have established a human-to-human transmission cycle are also included if the original pathogen transmission was considered climate-sensitive. CSIDs included in this review are:…”*

**Minor comments**

4. **Abstract**
   A summary of your exclusion criteria would be helpful. As it currently stands, I can’t quite understand the scope of your review.

- *Response*
- *We excluded articles published outside of the years 2010-2023, were published outside of sub-Saharan Africa, non-English articles, news articles or conference abstracts, articles that consider climate hazards in relation to barriers to healthcare facility access, articles about allergens or fungal diseases, and articles that did not apply or assess economics or data systems in the context of pandemic preparedness.*
- *To clarify the scope of the review in the abstract we have added a summary of the inclusion criteria from line 58: “Studies met inclusion criteria if they were published between 2010 -2023, were relevant to sub-Saharan Africa, were relevant to pandemic preparedness, a target CSID, and applied or assessed economic evaluations or data systems.” We have chosen to include a summary of inclusion criteria rather than exclusion criteria due to word count restraints, as we believe that the exclusion criteria only makes sense in conjunction with the inclusion criteria.*

5. I**ntroduction**
   Line 148: “we extended a conceptual framework.” Is this a conceptual framework that already exists and you extended it? Or something new you developed?

- *Response*
- *The conceptual framework is based on an existing framework defined by the WHO (WHO. Ebola and Marburg virus disease epidemics: preparedness, alert, control, and evaluation. 2014.). We extended it to illustrate links to economics and data systems for pandemic preparedness. This method has been clarified in the introduction from line 161: “To identify existing knowledge and gaps relevant to data system and economic studies for priority setting of CSIDs with pandemic potential, we adapted a conceptual framework based on four stages of pandemic preparedness identified by the WHO (18): Prevention (Stage 1) ….”*

6. Line 161: I don’t think intersectional approaches to analyses necessarily imply representation, or vice versa. Suggest clarifying language here

*Response*

- *We agree and have rephrased and dropped use of this term; line 106 now reads: “Investigating gendered impacts of pandemics can enable a more contextualised and thorough understanding of the aetiology of CSIDs and enable equitable responses tailored to men, women, boys, girls, and other gender identities (10).”*

7. Line 167-179: This reads like you’re trying to wrap quite a lot into a single project, and it makes it quite difficult for the reader to parse. I would harmonize these into a single list, and add clarity however you can (e.g., shorter sentences, a conceptual diagram, etc.).

- *Response*
- *We agree and have now streamlined these into a single list under the header “Objectives”, aligned to PRISMA-ScR guidelines, from line 182. Additionally, the language used within the objectives has been simplified to make it easier for the reader to understand.*

8. **Methods**
   Table 1: with AI tools (e.g., Google Translate and DeepL), it’s a bit harder to justify exclusion of articles based on language. Some text to justify this (or discuss it as a limitation) is needed.

- *Response*
- *We thank the reviewer for this insightful comment. This study was conducted as a rapid scoping review which necessitated limiting the inclusion of studies and reliance on additional methods and tools for the review (including e.g., AI tools for translation or synthesis). While we aimed to capture the majority of relevant studies within the timeframe available, we acknowledge that excluding articles based on language is a limitation as discussed in the limitations section from line 790.*

9. Also, the climate change inclusion criteria cell is really confusing as written—for instance you say articles that referred to infectious diseases impacted by climate change were included, but then you list variables defining climate change, not infectious diseases. Did you include all CSIDs with zoonotic potential, or just those with pandemic potential? Ideally this would be in list form (true for the whole table).

- *Response*
- *We thank the reviewer for bringing this to our attention. The inclusion process was for pathogens with pandemic potential defined by previously published reports (PHEIC and R&D pathogens), and compared with the CSID database, see response to comment 3 for further details. To address this comment in the manuscript, we have clarified the inclusion criteria in Table 1. “Climate change” has been refined to “Climate-sensitive infectious diseases” and the steps to identify CSIDs has been outlined, listing the final diseases included (see response to comment 3).*

10. Line 231: Independently screened the remaining abstracts?

- *Response*
- *Yes, now amended (line 254).*

11. Line 273: “Use data from data systems.” Was this intended to say “from multidisciplinary data systems”?

- *Response*
- *Yes, now amended (line 299).*

12. Lines 284-291: Is intersectionality relevant to all of the study types listed in the previous paragraphs? I guess I’m not sure how this makes sense for studies that fit the data systems: design category since those aren’t research studies.

- *Response*
- *As per response 6 above, the terminology related to gender analysis has been simplified and the term intersectionality has been dropped. However, we believe that the gender analysis remains relevant to the "Design" data system studies, as we aimed to assess whether and how gender considerations were incorporated into the production of data systems.*

13. Line 302: “the large volume of literature utilizing data from data systems.” I’ll say! Perhaps you want to be more specific here?

- *Response*
- *We thank the reviewer for this comment, and have subsequently clarified the updated definition and our reasoning behind the protocol amendment from line 325: “First, we refined the fourth abstract screening question relating to data systems by applying more stringent criteria to the data systems theme, as outlined in Table 1. Specifically, we wanted to capture studies that used data from established data systems, that describe the creation of new data systems, or that outline the interoperable use of multi-sectoral data systems. This amendment was required to streamline the screening process conducive to our rapid review timeframe.”*

14. Throughout: I notice you use the term “iteratively refine” a few times. It’d be helpful to have just a few extra words in these locations detailing how this was done, or at minimum what was the endpoint.

- *Response*
- *Clarification has been given across the three places where “iteratively refined” was used.*

15. **Results:**
    I’d suggest breaking down the results for Design data systems into subsections with subheaders, to make it easier to follow

- *Response*
- *PLOS GPH only allows for three heading levels, so breaking this down into more subsections would use a fourth level. However, one of the paragraphs surrounding levels of data sharing has been significantly reduced and integrated into a previous paragraph from line 457, which makes the section easier to follow. The sentence now reads: “The varying scopes of these data systems highlight the extent of data sharing for CSID outbreak detection and response, spanning local (36, 46), national (32, 38, 40, 43, 46, 47, 49), regional (33, 34, 39, 41, 44), and international levels (30, 31, 33, 35, 37, 42, 45, 48).”*

16. Lines 498-513: this seems to be more of a gender analysis than analysis of intersectionality. What about other marginalized identities, besides gender? Also, I assume all of these studies treated gender as a binary, but it’d be worthwhile to reflect on that.

- *Response*
- *We agree, please see response to comment 6 above. In addition, we have added comments regarding treating gender as a binary in i) the methods (line 318), and ii) the limitations (line 811) section of the discussion.*

17. Line 522: I am surprised that you only found 8 studies investigating the cost effectiveness of COVID-19 measures in sub-Saharan Africa, and only 2 for Ebola also seems really small. Can you elaborate on this—where (in terms of your exclusion criteria) did most of these get weeded out?

- *Response*
- *Relevant literature was likely omitted by not including grey literature, which may have included policy reports that describe the production and use of economic evaluations, including those based on COVID-19 and Ebola. This is discussed in the limitations section from line 791: “We did not conduct a grey literature search due to the expedited timelines of the rapid scoping review, likely omitting relevant literature including governmental policy reports that may describe governmental data-system and economic evaluation use.”*
- *We were expecting a limited number of economic studies from peer reviewed literature on COVID-19 and Ebola based on previous analyses that have been conducted, which shows that the majority of literature has been based on other diseases/indications. In general, the health economic literature centred on Africa is dominated by HIV/AIDs and maternal and child health (*[*Meng et al., 2022*](https://publichealthinafrica.org/index.php/jphia/article/view/432)*) irrespective of study type.*
- *The small number of studies found is reflected upon in the discussion from line 706: “The limited number of economic studies, along with their concentration on COVID-19 and Ebola and primarily conducted in South Africa, suggests a broader deficiency in capacity and technical expertise for conducting similar evaluations across the rest of the WHO AFRO region and for other CSIDs”.*
- *We hope this provides additional context for the low number of studies included in our review, and highlights the challenges in identifying economic studies specifically focused on CSIDs in sub-Saharan Africa.*

18. Lines 573-586: I’m surprised it was pretty much all Ebola and COVID-19, especially as COVID-19 isn’t really a CSID (on this note, I question the decision to include it since it dominates so much of your review). It’d be helpful to see a list of the diseases/pathogens you included—I assume vectorborne diseases were excluded?

- *Response*
- *See response to comment 3 which justifies our inclusion of COVID-19 as a CSID in this review.*
- *We have changed the list of CSIDs in Table 1 to bullet point format to clearly outline which CSIDs were included, which were: Influenza A, Ebola Virus Disease, Marburg Virus Disease, Zika Virus Disease, Severe Acute Respiratory Syndrome (SARS), Middle East Respiratory Syndrome (MERS), Coronavirus Disease 2019 (COVID-19), Crimean-Congo Haemorrhagic Fever, Lassa Fever, Nipah Virus Disease, Henipavirus Disease, Rift Valley Fever, Mpox, Disease X.*
- *VBDs such as Rift Valley Fever and Zika were included as Target CSIDs as they were on the WHO R&D and/or PHEIC lists, and were also characterised as climate-sensitive according to the Mora (2022) review.*

Reviewer #2:

The paper provides an overview of research and points towards research gaps for a pertinent issue: the role of data systems and economics in decision-making for climate-sensitive diseases. The paper is generally well-written and presented. Some comments to improve the manuscript further:

1. line 71, p.3: Temperature and rainfall are not climate hazards. You can rephrase this to "...the frequency and intensity of climate hazards (e.g., droughts, floods, and heatwaves)".

- *Response*
- *We thank the reviewer for highlighting this, we have rephrased as suggested on line 81.*

2. lines 78-79, p.4: It would be good to specify the years covered by your review to contextualise the eight viral diseases declared as PHEICs by the WHO.

- *Response*
- *We thank the reviewer for this constructive comment. The review covered the years 2010 – 2023, which included the first PHEIC to be declared after the 2009 – 2010 outbreak of H1N1* [*(Wilder-Smith & Osman, 2020)*](https://pmc.ncbi.nlm.nih.gov/articles/PMC7798963/)*. We have clarified this in the manuscript based on the comment from line 96 to* “*This review covered the years 2010–2023, encompassing the period of all PHEIC declarations by WHO including Influenza and COVID-19, up to 2023*.”

3. lines 87-95, p.4: Similar to the start of the Abstract and line 159 on p.7, a broader definition of vulnerable populations was initially given but later only gender differences were considered. This is also seen in the "Gender-sensitivity of data system studies" section starting on p.23. If this is the case, I would suggest making the focus on the gender-lens more explicit in the Abstract and Introduction, and adding a sentence or two to support this focus over other vulnerable populations.

- *Response*
- *We thank the reviewer for raising this important issue, we agree and have dropped use of the term “intersectionality”. Instead, the gender analysis is described as “application of a gender lens” in the methods (line 310), and results (lines 544 and 658). We have streamlined the focus of the analysis on gender and not other social determinants of health by highlighting the need to identify and respond to gendered needs during pandemics in the introduction on line 108: “Investigating gendered impacts of pandemics can enable a more contextualised and thorough understanding of the aetiology of CSIDs and enable equitable responses tailored to men, women, boys, girls, and other gender identities (10).”*

4. Objectives and Research questions, p.7-8: It is unclear to me why there is a need to separate these two sections. The points are similar and can be combined under one section.

- *Response*
- *We agree and have now streamlined these into a single list under the header “Objectives”, aligned to PRISMA-ScR guidelines, from line 182.*

5. Table 1, p.9: Are the stages mentioned on p.7 in line with WHO Pandemic Preparedness Framework mentioned under "Pandemic Preparedness"?

- *Response*
- *Yes, the stages of pandemic preparedness are the same as outlined in the introduction. The language in Table 1 to describe the stages has been amended to better reflect this: “Articles that assessed preparedness that can be contextualised within one of the four stages of adapted conceptual framework (S1 Fig), including Prevention (Stage 1); Detection (Stage 2); Response (Stage 3); and Evaluation (Stage 4).”*

6. Table 1, p.10: "AMR" is not defined.

- *Response*
- *Amended (Table 1).*

7. lines 272-273, p.14: Two different spellings of "multi-disciplinary" were used.

- *Response*
- *Amended for consistency.*

8. lines 299-301, p.15: Is this definition of data systems the same as that mentioned in Table 1?

- *Response*
- *Thank you for bringing this to our attention, we had included the previous definition of data systems in Table 1. This has been updated in Table 1 and greater clarification of the need for this amendment has been provided in the “protocol amendments” section from line 327: “First, we refined the fourth abstract screening question relating to data systems by applying more stringent criteria to the data systems theme, as outlined in Table 1. Specifically, we wanted to capture studies that used data from established data systems, that describe the creation of new data systems, or that outline the interoperable use of multi-sectoral data systems. This amendment was required to streamline the screening process conducive to our rapid review timeframe.”*

9. line 302-303, p.15: Can you clarify what you mean by "a pilot of data extraction in duplicate"?

- Response
- *We had originally intended for two reviewers to extract data from the same papers and compare them, to investigate whether the same data would be extracted and subsequently clarify the data extraction form if required. Unfortunately that was not possible due to the rapid review timelines. To address this comment, we have clarified the description of the protocol amendment on line 330 to “Second, we did not conduct a pilot of data extraction in duplicate, where two reviewers would independently extract data from the same paper to test the clarity and reproducibility of the form”.*

10. Table 2, p.26-30: The "Aims" column seems unnecessary because all the details are captured in the other columns and all the papers are CEAs. I would suggest removing this column.

- *Response*
- *We thank the reviewer for this constructive comment, we have adopted this suggestion and have removed the “Aims” column to streamline the table.*

11. line 567, p.31: There is an extra space between "for" and "costing". There is also an extra space between "in" and "duplicated" on line 595 of p.33, and between "gender" and "-blind" on line 621 of p.37.

- *Response*
- *Corrected throughout.*

12. Table 3, p.32: The "Aims" column can be removed. For consistency with Table 2, you can add a "Intervention/comparators" column too.

- *Response*
- *Adopted. We have removed the “Aims” column in line with response to comment 10, and have added an intervention/comparator column.*
