# Supplementary material for: Gaps and opportunities for data systems and economics to support priority setting for climate-sensitive infectious diseases in sub-Saharan Africa: A rapid scoping review
Source: PLOS Glob Public Health. 2025 Jun 11;5(6):e0003814. doi: 10.1371/journal.pgph.0003814 (PMC12157337; doi:10.1371/journal.pgph.0003814)
Supplement: Attachment — Submitted filename: Response to reviewer comments CSID PP R2_vF.docx [file pgph.0003814.s010.docx]

# to reviewer comments

Reviewer #1: Thank you for your thoughtful response to my comments. A lot is improved in this version; several major comments are below. My primary concern now has to do with your search terms being perhaps too restrictive (or your objectives need to be re-clarified).

*We sincerely thank the reviewer for their insightful comments. Responses to individual comments are provided below.*

*To provide additional context, the overarching themes for this study – economics and data systems related to climate-sensitive infectious diseases and pandemic preparedness, and the inclusion of a gender lens – were specifically identified by the funder. These topics are well characterised research priorities due to the funder’s long and active engagement in supporting research designed to strengthen health policy through data systems development in the African region.*

*While the funder identified these thematic areas, the study design and methodology were wholly and independently determined by our research team. We opted to conduct a single review, rather than separate reviews on data systems and economic evaluations, as we view these themes as interconnected within the broader perspective of priority-setting processes. Given constraints on the timeline that we had to undertake the review, we determined that a single rapid scoping review with shared objectives and a unified search strategy was the most effective approach. This allowed us to systematically identify relevant literature across both domains while maintaining consistency in the search methodology and ensuring a comprehensive synthesis of findings. While we acknowledge that this approach may have impacted the breadth of our scoping search, we are confident that the trends found in our review are credible and in line with the recent literature.*

*We hope this clarification provides additional context to the manuscript and our responses below. Please note that references to line numbers in this document refer to the marked up version of the manuscript.*

**Major comments:**

1. Positionality statement:
   These are typically at the start of papers, and usually look more like this: <https://pmc.ncbi.nlm.nih.gov/articles/PMC11007728/>. I’m also concerned about the statement re: foregrounding the perspectives of African researchers when neither the first author nor senior author is African.

   That being said, I don’t think every paper needs a positionality statement, nor is it required by this journal (to my knowledge). Furthermore, I appreciate the authors’ efforts to be responsive to this comment. Really, it’s the bibliometric analysis/temporal publication trends that to me is the trigger for needing a positionality statement.

   I’d suggest removing the positionality statement and de-emphasizing the temporal publication trends part of your bibliometric analysis by focusing instead on the geographical publication trends, and including authorship trends as a sentence at the end of that paragraph (remove the temporal publication trends paragraph). Then when you discuss in the Discussion section, add a sentence acknowledging that neither the lead author nor senior author on this paper are African, however….(add the justification currently in the positionality statement).

   If you do want to keep temporal publication trends in as a separate paragraph in the methods and results, then I think you need to re-vamp your positionality statement to be more in line with the one I shared.

*We appreciate the feedback regarding the positionality statement. We have revised the manuscript to de-emphasise the bibliometric analysis, particularly the temporal publication trends, and have removed the positionality statement accordingly.*

*Specifically, we have:*

- *Focused on the geographical publication trends and incorporated authorship trends as a sentence at the end of that section as on line 349: “The frequency of publications increased between 2010 and 2023, with authorship trends indicating a growing involvement of researchers based in African institutions in CSID research. However, mixed institutional research teams remain dominated by institutions based outside the continent up to 2023.” We have also removed the discussion of the author affiliations from the Discussion section.*
- *Moved the CSID publication trends to the section previously known as “Topic Mapping” but now titled “Climate-sensitive infectious diseases” from line 367: “From 2010 to 2023, COVID-19, Ebola, Influenza, and Rift Valley fever were the most studied target CSIDs (Fig 3a), with topic mapping revealing broad coverage across all CSIDs in data system studies but much more limited coverage in economic studies (Fig 3b).”*
- *Acknowledged in the discussion that neither the lead nor senior author is African in the limitations on line 753: “Finally, neither the lead nor senior author is African (latter is African resident), and we recognise that interpretations may be shaped by training, institutional affiliations, and lived experiences. To address this, we drew on the expertise of a multidisciplinary, international research and authorship team predominantly composed of African researchers. All authors collaboratively contributed to the review design, analysis, and interpretations, including on gender equity components.”*

Introduction

2. Line 89-90: This is absolutely true, but I’m not sure that it makes sense to link this to economic analyses. Addressing systematic oppression is critical to resolving health disparities and climate vulnerability, but it’s not necessarily cost-effective (i.e., justice is not necessarily advanced by linking it to economic arguments).

*We agree that addressing systematic oppression and gender inequalities is essential to mitigating health disparities and climate vulnerability. Our aim is not to suggest that economic efficiency should dictate justice efforts but rather to encourage economic analyses to account for gendered vulnerabilities more explicitly. The introduction aims to highlight that economic analyses often underpin priority-setting processes, and that these evaluations rely on data systems that may not adequately capture gendered data.*

*By examining whether gender is considered within economic and data-system domains, we can identify potential biases in priority setting processes. This could help ensure that resource allocation and policies are informed by a more comprehensive understanding of differential risks and needs across genders.*

*This has been addressed in the manuscript on line 92: “Given that economic evaluations and data systems often inform priority setting in policy responses, it is important to assess whether gendered vulnerabilities are adequately considered within these frameworks. If gender is overlooked in these domains, critical disparities may go unrecognized, limiting the effectiveness and equity of interventions.”*

3. Line 158-164: Your objectives don’t mention the gender analysis, which leaves me still a bit confused about the point and scope of this paper. If the gender analysis is a major piece, then I would make that clearer in your intro (and possibly call it out as a sub-objective)

*We thank the reviewer for bringing this to our attention. We believe the gender analysis forms a major part of this manuscript and is important to keep in. Therefore the gender component has been strengthened in the introduction with additional text:*

- *See response to comment 2 above.*
- *Line 131: “Recent outbreaks, such as the 2014 Ebola outbreak in West Africa (16) and more recently with COVID-19 globally, illustrate the long-lasting economic and health consequences of pandemics.* *These impacts are not experienced equally across genders, with women and other marginalised groups facing disproportionate economic losses, as seen during the COVID-19 pandemic (17).”*
- *Line 146: “Priority setting is therefore facilitated by having fit-for-purpose (and cost-effective) data systems that capture contextualised information on epidemiology, vulnerable groups, resource use, gender disparities, and other relevant socioeconomic characteristics.”*
- *We have also reintroduced an objective: Line 171: “Understand the extent to which gender considerations are incorporated into economic analyses and data system studies related to pandemic preparedness of CSIDs.”*

Results

4. Lines 460-467: Might be worth reflecting on the finding in lines 448-449 (inclusion of vulnerable groups in participatory surveillance)

*These referenced line numbers don’t quite correlate with our version of the manuscript, however we believe this comment relates to a gender-specific study that included women as key stakeholders in a surveillance system identified in our review as a data system ‘Usage’ study. This study acknowledges the important role of women in disease detection despite their frequent under-representation and provides an example of inclusion of vulnerable groups in participatory surveillance – an evidence gap identified by our review of studies, which had the objective of addressing equitable data system design.*

*We have now addressed this on line 492: “The authors of the gender-specific study included women as key stakeholders in a surveillance data system, acknowledging their important role in disease detection despite their frequent under-representation (37). This study addresses the evidence need for participatory approaches to ensure the inclusion of vulnerable groups in data collection (identified in reference 56).”*

*We welcome further clarification in case we have misinterpreted this comment, and the line numbers referenced.*

5. Lines 474-477: I’m honestly quite shocked there were no economic evaluation studies on RVF or influenza. I’m thinking this comes down to including “climate change” and “pandemic preparedness” in the search terms—once diseases have been identified as a CSID with pandemic potential, is it necessary to require that climate change and/or pandemic preparedness appears in the text or in a MeSH term? I think you’re finding “false positive” evidence gaps because of this.

   I think it’s important clarify whether your goal is to characterize the evidence among papers that “self-identify” as relevant to climate change and pandemic preparedness, or merely to characterize the available evidence. If the latter, I don’t think your search terms are well-suited. Effectively, I think it might have been better to have different search terms for the different aims of your study (bibliometric analysis, data systems, economic evaluations, priority settings, etc.)

*Thank you for this insightful comment. We appreciate the concern about potential "false positive" evidence gaps due to our search strategy. While we worked with an experienced information specialist to develop a search strategy that aligned with the study’s scope, we acknowledge that it may not have been as comprehensive as it could have been, particularly for each sub-theme explored when considered in isolation. Specifically, using the same search strategy for both data systems and economic studies might have led to a loss of specificity for these domains. If separate search terms had been employed for these distinct themes, it is possible that we would have captured more targeted results.*

*However, we believe the trends captured in our manuscript are consistent with and do reflect trends in the literature. For example, the lack of economic studies we identified is reflected in the paper* [*Meng et al., 2022*](https://publichealthinafrica.org/index.php/jphia/article/view/432)*, who assessed health economic research trends in Africa from 1991 to 2020. Only 7 out of 178 health economic papers included in their review assessed infectious disease trends. Within infectious diseases the authors included malaria and pneumonia, both of which were not included in our review, as well as vector-borne diseases, of which we only included Zika. Furthermore, we identified current data sharing gaps across human, animal, and environmental domains which was also highlighted in the* [*WHO Future Surveillance Report (2023)*](https://iris.who.int/bitstream/handle/10665/374992/9789240080959-eng.pdf) *emphasises the necessity to address this gap to prepare for future pandemics. The WHO report also highlights the importance of participatory and community-based surveillance that leverages digital technologies for surveillance, which was also identified as an gap from the studies included in our review.*

*Regarding the inclusion of “climate change” and “pandemic preparedness” search terms, we aimed to capture studies explicitly addressing these themes while also ensuring that relevant studies were not excluded solely due to terminology. For climate change, we included a broad range of search terms related to environmental exposures, such as weather, hydrometeorological hazards, and air quality. Similarly, for pandemic preparedness, we incorporated search terms covering communicable diseases with pandemic potential, WHO R&D Blueprint Pathogens, PHEIC-listed diseases, and related terms such as “epidemics” and “disease outbreaks.” Importantly, studies did not need to explicitly use the terms “pandemic preparedness” or “climate change” to be included. We used a conceptual framework (Appendix 2, S1 Fig) to map studies to different stages of pandemic preparedness, meaning that studies addressing disease transmission, risk factors, or health systems were included even if they did not self-identify under these categories. Results of this can be found in the appendix 3, and is referred to in the manuscript on line 261: “Studies were also mapped to each stage of pandemic preparedness, with these results provided in the supplementary information (S4 Fig).” For example, a study that aimed to identify ecological risk factors of RVF outbreaks in eastern and central Kenya and to produce a spatially explicit risk map using data systems (*[*Mosomtai, et al., 2016*](https://pubmed.ncbi.nlm.nih.gov/26996461/)*) was included under Stage 1 of the pandemic preparedness framework.*

*Regarding the absence of economic evaluations on RVF, it is important to note that RVF is included in the WHO R&D Blueprint list precisely because there are significant gaps in expertise and research. We believe the absence of economic studies in our findings therefore reflect a genuine gap in research rather than an artifact of the search strategy.*

*The final search strategy, adapted for each database, is available in Appendix 1 in this document on* [*the LSHTM Data Compass*](https://dx.doi.org/10.17037/DATA.00003769) *(referenced in the manuscript).*

6. Line 528: I think by virtue of your search terms, the priority setting papers would have to discuss either economic evaluations or data systems—that means you’ll have a biased sample and won’t see papers that use other means for priority setting.
    *Thank you for this thoughtful comment. Our intention was to focus specifically on priority-setting papers that were situated within an economic context, as our study aimed to assess how economic evaluations and data systems inform decision-making for CSIDs. We recognise that priority setting can take many forms, including deliberative processes, expert consensus methods, and burden-of-disease approaches. However, these were beyond the scope of this review, which was designed to examine economic considerations in priority setting.*

Discussion

7. Line 613-614: Again, I think part of this is an artefact of your search terms: I don’t think you’ll find a lot on RFV if you require papers to refer to pandemic preparedness, but that doesn’t mean there’s gap in capacity or expertise

*See response to comment 5 above.*

**Minor comments:**

Abstract

8. Line 42: should States be capitalized?

*No, amended.*

Methods

9. Table 1: are the inclusion criteria for health economic theme and data system theme restricted to the CSIDs listed in the row above?

*Yes, this has been clarified on line 216: “Articles were included if relevant to a stage of pandemic preparedness AND a target CSID AND data systems OR economic analyses (see S1 Table).”*

10. Lines 209-211: I’m confused about what happened between the abstract review and the full text review. Were some of the abstracts excluded? Currently, it reads as if two people read abstracts, then read full texts, but I’m not sure why they would do that.

*Yes, articles were excluded at the abstract stage. A sentence has been added to line 221 clarifying this: “Articles were excluded if the abstract did not meet the inclusion criteria.”*

11. Lines 237-243: The bibliometric analysis needs to be mentioned in the introduction. It’s confusing to come across it for the first time in the Methods section.

*This has been incorporated into the first objective on line 163 as “Map key themes that describe how economic analyses and data systems support priority setting during pandemic preparedness of CSIDs in sub Saharan Africa, using bibliometric analysis, topic mapping and narrative synthesis.”*

Results

12. Lines 351-353: Suggest re-wording to: “Five CSIDs dominated the data system literature: Rift Valley fever (22 studies), Ebola virus disease (14 studies), influenza (12 studies), COVID-19 (12 studies), and Lassa fever (8 studies)” as it’s easier for the reader to parse

*We have rephrased as suggested on line 374.*

13. Line 389: capitalize One Health

*Amended.*

14. Line 392: replace “extent” with “range,” as the second part of the sentence doesn’t tell me that data sharing is extensive, it just tells me the extent varies

*Amended.*

15. Line 399: What about environmental surveillance?

*We have revisited our data extraction form and added an additional gap regarding environmental surveillance on line 431 taken from* [*Liebenberg et al., 2017*](https://www.sciencedirect.com/science/article/abs/pii/S0006320716301707)*: “In the environmental sector, an absence of local stakeholder involvement was reported, which resulted in environmental monitoring being an isolated academic exercise driven by national and international stakeholders (34)”.*

Reviewer #2: The authors have addressed all my comments.

1. Please note however, that there is a typo on line 86 on page 4: "declarations the WHO" should be "declarations by WHO".

*Thank you for bringing this to our attention. Amended.*

# Appendix

## Appendix 1: Complete search strategy

The full search strategy is included for OvidSP Medline ALL, the full search strategy for all other databases can be found at [the LSHTM Data Compass](https://dx.doi.org/10.17037/DATA.00003769).

Final search strategies

1 Search methodology

A draft search strategy was compiled in the OvidSP Medline ALL database by an experienced information specialist. The search strategy included strings of terms and synonyms to reflect the following concepts:

- Concept 1: Climate change related environmental exposures. This includes weather, hydrometeorological hazards and air quality.
- Concept 2: communicable diseases with pandemic potential
- Concept 3: Africa
- Concept 4: data systems or economic evaluation. This includes health technology assessments and priority setting.

The search was limited by date to items published 2010-present.

The search strategy was refined with the project team until the retrieved results reflected the scope of the project. Once agreed, the draft search was adapted for each database to incorporate database-specific syntax and controlled vocabularies.

Full details of the search strings used for each database can be found in the appendix.

2 Sources

Search strategies for were run on the following bibliographic databases on 27 November 2023:

* OvidSP Medline ALL, 1946 to November 22, 2023;

* OvidSP Embase Classic+Embase, 1947 to 2023 November 22;

* OvidSP Global Health, 1910 to2023 Week 46;

* OvidSP Econlit, 1886 to November 16, 2023

* EBSCOhost Africa-Wide Information, complete database;

* Clarivate Analytics Web of Science, data updated 2023-11-24

The following databases which were searched simultaneously:

- Science Citation Index Expanded (SCI-EXPANDED), 1970-present;
- Social Sciences Citation Index (SSCI), 1970-present;
- Arts & Humanities Citation Index (SSCI), 1975-present;
- Conference Proceedings Citation Index-Science (CPCI-S), 1990-present;
- Conference Proceedings Citation Index-Social Science & Humanities (CPCI-SSH), 1990-present;
- Emerging Sources Citation Index (ESCI), 2018-present.

3 Information management

All results from the database searches were exported and uploaded to EndNote 20 software. Duplicates were identified and removed using the method described on the LAORS blog.(a) The order the database results were uploaded is included in the appendix.

4 Results

Details of results from each database are shown in the table below.

Database name

Results

Results after duplicates removed

Medline ALL

2619

2611

Embase Classic+Embase

4475

2430

Global Health

2557

945

Econlit

75

53

Africa-Wide Information

1605

393

Web of Science Core Collection

2921

787

Total

14,252

7219

5 Appendix: Search strategies

This appendix provides full details of all search strings used for bibliographic databases, with dates and number of references returned and notes explaining any unusual search techniques or syntax.

5.1 OvidSP Medline ALL

Database name: Medline ALL

Database platform: OvidSP

Dates of database coverage: 1946 to November 22, 2023

Date searched: 2023-11-27

Number of results: 2619

EndNote deduplication order: 1

Results after duplications removed: 2611

Search strategy notes

Search lines ending in a ‘/’ are subject heading searches.

Search lines beginning ‘exp’ are exploded subject heading searches.

Two-letter codes at the end of search lines designate the fields to search. Fields codes used are:

TI: title

AB: abstract

adjn searches for words within n words of each other.

or/x-y combines search sets in the range x-y with Boolean operator OR.

* is used for truncation of words.

? finds 0 or 1 character in that placeholder (optional wildcard).

words in square brackets […] are comments and are ignored.

\#

Search terms

Results

1

exp Human Activities/ and (ecosystem/ or exp ecology/ or environment/)

6743

2

((environment* or ecosystem* or ecolog*) adj3 (change* or variab* or disrupt* or hazard* or impact* or vulnerab* or function* or footprint* or function* or extreme or exposure or exposed or expose or sustainab*)).ti,ab.

179102

3

((environment* or global or planet* or ecolog*) adj1 (sustainab* or regenerat*)).ti,ab.

7376

4

(climat* adj3 solution*).ti,ab.

372

5

exp Forests/

17179

6

exp Tundra/

814

7

Wetlands/

11227

8

exp Fresh Water/

82232

9

Wilderness/

365

10

Rivers/

36499

11

Parks, recreational/

2589

12

(forest* not (random survival forest or forest plot*)).ti,ab.

103842

13

(tree? not decision tree).ti,ab.

153905

14

woodland?.ti,ab.

4220

15

(grass not (grass-carp or grass-root?)).ti,ab.

26263

16

(greenspace? or green-space?).ti,ab.

3126

17

green-infrastructure.ti,ab.

543

18

park?.ti,ab.

26928

19

tundra.ti,ab.

1772

20

permafrost.ti,ab.

1885

21

wetland?.ti,ab.

15903

22

fresh-water.ti,ab.

7147

23

(bluespace? or blue-space?).ti,ab.

297

24

lake?.ti,ab.

48756

25

pond?.ti,ab.

14191

26

(dam or dams or reservoir*).ti,ab.

106560

27

river?.ti,ab.

81106

28

wilderness.ti,ab.

1300

29

desert?.ti,ab.

16047

30

"the bush".ti,ab.

3724

31

coast*.ti,ab.

77270

32

beach*.ti,ab.

10225

33

Biodiversity/

42039

34

Endangered species/

5473

35

Introduced species/

7285

36

Extinction, biological/

4343

37

biodivers*.ti,ab.

38890

38

biological divers*.ti,ab.

2605

39

(extinct* adj2 (rate? or species)).ti,ab.

3418

40

((wildlife or species) adj2 (protect* or declin* or abundan* or rich*)).ti,ab.

28340

41

protected area?.ti,ab.

5674

42

biosphere.ti,ab.

5186

43

exp Climate Change/ or Global Warming/

31117

44

(exp Weather/ or exp Climate/) and exp Disasters/

16103

45

((climate or climactic) adj3 (change? or changing or variab* or disrupt* or hazard* or impact* or vulnerab* or function* or extreme or catastroph* or emergenc* or shift* or dynamic* or sensitiv* or sustainab* or anomal*)).ti,ab.

68763

46

((hydrometeorolo* or atmosphere* or hydrologic* or ocean* or weather) adj3 (change* or variab* or disrupt* or hazard* or extreme or exposure or exposed or expose or phenomen* or sustainab* or anomal*)).ti,ab.

10186

47

extreme weather.ti,ab.

2203

48

natural hazard?.ti,ab.

943

49

global tipping point?.ti,ab.

5

50

exp Temperature/

454044

51

Ice Cover/

2897

52

Permafrost/

458

53

((global or planet* or world or climat* or earth or worldwide or world-wide or ambient air or ocean*) adj1 (heating or warming or temperature*)).ti,ab.

20604

54

((temperature? or cold or heat or hot or ambient air or relative humidity or thermal) adj4 (extreme or elevate* or irregular or intense or reduced or pattern* or anomal* or global)).ti,ab.

55997

55

(ENSO or el nino-southern oscillation).ti,ab.

1441

56

thermal threshold?.ti,ab.

931

57

thermal stratification.ti,ab.

347

58

((arctic or antarctic or pack-ice or ice-sheet or glaci* or snow*) adj3 (shrink* or decrease* or melt*)).ti,ab.

1814

59

(heatwave? or heat-wave?).ti,ab.

3584

60

(coldwave? or cold-wave?).ti,ab.

154

61

(thermal environ* or heat-island*).ti,ab.

3197

62

Floods/

4100

63

Droughts/

12452

64

((sea-level or sea level) adj5 (rise? or rising or change? or changing)).ti,ab.

2572

65

cloud cover.ti,ab.

686

66

((rain* or precipitation) adj4 (extreme or elevate? or elevating or irregular or intense or reduced or pattern* or anomal*)).ti,ab.

5141

67

flood*.ti,ab.

23397

68

water current?.ti,ab.

498

69

water stress.ti,ab.

5605

70

drought?.ti,ab.

33188

71

Cyclonic Storms/

3070

72

tropical cyclone?.ti,ab.

695

73

hurricane?.ti,ab.

4635

74

storm*.ti,ab.

23358

75

((tide or tidal or sea or ocean*) adj2 (surge or surges)).ti,ab.

66

76

((ocean* or sea or marine) adj2 (ph or acidification or heating or warming or change* or variab* or extreme or expand or expansion or sustainab*)).ti,ab.

7333

77

windstorm?.ti,ab.

106

78

Landslides/

282

79

Wildfires/

1252

80

landslide?.ti,ab.

1223

81

(wildfire? or wild-fire?).ti,ab.

3657

82

((fire? or burnt or inferno or conflagration) adj2 (change* or variab* or extreme or expand or expansion or intense or elevate? or increas*or catastroph* or emergenc* or shift* or dynamic* or frequen* or more)).ti,ab.

2269

83

Water Supply/

34756

84

exp Groundwater/

14457

85

(water adj3 footprint).ti,ab.

622

86

(global freshwater or global fresh-water).ti,ab.

152

87

global water cycle.ti,ab.

65

88

((freshwater or water) adj1 (provision or provid* or supply or sustain*)).ti,ab.

12936

89

(groundwater or ground-water).ti,ab.

29399

90

aquifer*.ti,ab.

9669

91

water table.ti,ab.

1550

92

watershed.ti,ab.

11701

93

(water adj4 (resource* or conserv* or security or availab* or scarcity or poverty or stress or overconsumpt* or over-consumpt* or save or saving or sustainab*)).ti,ab.

35796

94

hydrological flow*.ti,ab.

57

95

soil erosion/

136

96

(land supply or land supplies).ti,ab.

37

97

"land use change?".ti,ab.

4114

98

(habitat adj2 (loss or fragment* or disturb* or alter or altered or destruct*)).ti,ab.

5735

99

soil erosion.ti,ab.

2259

100

soil quality.ti,ab.

2890

101

land cover change*.ti,ab.

1193

102

nutrient run-off.ti,ab.

12

103

deforestation.ti,ab.

3607

104

land degradation.ti,ab.

726

105

"slash-and-burn".ti,ab.

162

106

desertificat*.ti,ab.

1130

107

Greenhouse Effect/

6181

108

Greenhouse gases/

2568

109

ozone depletion/

71

110

(greenhouse effect* or green-house effect*).ti,ab.

868

111

(greenhouse gas* or green-house gas*).ti,ab.

14650

112

(("co2" or carbon or methane or ozone or nitrous oxide) adj5 (elevat* or emission* or rise or increas* or decreas* or reduc* or neutral or offset* or sustainab*)).ti,ab.

83726

113

((global or planet* or world or earth or worldwide or world-wide) adj2 ("co2" or carbon or methane or ozone or nitrous oxide)).ti,ab.

4328

114

(emission* adj5 (elevat* or rise or increas* or decreas* or reduc* or offset* or sustainab*)).ti,ab.

31993

115

carbon footprint/

1157

116

(carbon adj3 footprint).ti,ab.

2287

117

(de-carbon or de-carboni* or decarbon or decarboni*).ti,ab.

1072

118

exp water pollution/

32318

119

(water adj2 (pollut* or contamina*)).ti,ab.

26337

120

water quality.ti,ab.

27893

121

exp waste products/

101598

122

waste.ti,ab.

96354

123

rubbish.ti,ab.

299

124

trash.ti,ab.

1149

125

garbage.ti,ab.

2086

126

fly-tipping.ti,ab.

7

127

dump*.ti,ab.

8749

128

sewage.ti,ab.

25555

129

(faeces or faecal or feces or fecal).ti,ab.

141637

130

excrement.ti,ab.

601

131

slurry.ti,ab.

7817

132

"Environmental Restoration and Remediation"/

9486

133

cleanup?.ti,ab.

6957

134

environmental remediation?.ti,ab.

3408

135

environmental restoration.ti,ab.

257

136

pollution remediation?.ti,ab.

332

137

Sanitation/

8375

138

sanitat*.ti,ab.

14666

139

or/1-138

1863953

140

disease outbreaks/

92172

141

epidemics/

14144

142

pandemics/

123641

143

pandemic*.ti,ab.

220039

144

epidemic*.ti,ab.

138232

145

(public health emergenc* or PHEIC).ti,ab.

6497

146

(disease adj1 spread*).ti,ab.

4421

147

outbreak*.ti,ab.

141850

148

(climat* adj3 (infect* or communicab* or diseas*)).ti,ab.

1578

149

(geograph* adj3 diseas*).ti,ab.

1732

150

Influenza A Virus, H1N1 Subtype/

17696

151

"h1n1".ti,ab.

19933

152

Hemorrhagic Fever, Ebola/ or Ebolavirus/

7929

153

(haemorrhagic fever or hemorrhagic fever or ebola or ebolavirus).ti,ab.

20712

154

Zika Virus/ or Zika Virus Infection/

8188

155

zika.ti,ab.

11397

156

Severe acute respiratory syndrome-related coronavirus/ or coronavirus infections/ or severe acute respiratory syndrome/

52241

157

Middle East Respiratory Syndrome Coronavirus/

2064

158

COVID-19/ or SARS-CoV-2/

253017

159

(sars* or severe acute respiratory syndrome).ti,ab.

133017

160

(mers* or middle east respiratory syndrome).ti,ab.

11186

161

(covid-19 or ("2019" adj2 (coronavirus* or ncov)) or wuhan coronavirus).ti,ab.

340557

162

Hemorrhagic Fever, Crimean/

1289

163

Hemorrhagic Fever Virus, Crimean-Congo/

1161

164

((crimean or congo) adj2 (hemorrhagic or fever or virus)).ti,ab.

2057

165

Lassa Fever/ or Lassa virus/

1226

166

(lassa adj1 (fever or virus or infect*)).ti,ab.

1549

167

exp Henipavirus/ or Henipavirus Infections/

1010

168

(nipah or henipav*).ti,ab.

1517

169

Rift Valley fever virus/ or Rift Valley Fever/

1671

170

rift valley fever.ti,ab.

2308

171

(disease x not x-linked).ti,ab.

199

172

or/140-171

711536

173

exp Disaster planning/

15962

174

exp Communicable Disease Control/

419761

175

exp Technology Assessment, Biomedical/

12229

176

exp Health Planning/

375376

177

exp Regional Health Planning/

40824

178

exp Costs/ and Cost Analysis/

51652

179

exp Economics/

667627

180

exp Evidence-Based Practice/

94003

181

decision making/

105195

182

clinical decision making/

14704

183

health equity/

3914

184

exp records/

303064

185

exp algorithms/

445136

186

data systems/

270

187

database management systems/

7738

188

electronic data processing/

13420

189

exp computer security/

9750

190

exp data science/

824

191

exp data management/

1576

192

exp data mining/

11312

193

data compression/

2683

194

exp information management/

238612

195

(preparedness or ).ti,ab.

2526572

196

(prevent* adj1 control).ti,ab.

2115

197

((infection or disease* or measure*) adj1 control).ti,ab.

112658

198

(health technology assessment or HTA).ti,ab.

7793

199

(economic* or economy or economies).ti,ab.

411073

200

(macroeconomic* or macro-economic*).ti,ab.

1984

201

(priorit* adj1 (health* or set* or area*)).ti,ab.

15938

202

(resourc* adj1 allocat*).ti,ab.

15963

203

(resourc* adj3 distrib*).ti,ab.

4127

204

logistic*.ti,ab.

480298

205

surge capacit*.ti,ab.

753

206

((health* or resourc*) adj3 ration*).ti,ab.

3126

207

cost-effectiv*.ti,ab.

178985

208

(cost-benef* adj1 analys*).ti,ab.

5279

209

(cost-utilit* adj1 analys*).ti,ab.

3982

210

(cost or costs).ti,ab.

728342

211

(financ* or fiscal*).ti,ab.

154095

212

marginal analys*.ti,ab.

288

213

(benefit* adj2 cost*).ti,ab.

25566

214

(evidenc* adj2 (assess* or apprais* or eval*)).ti,ab.

24563

215

(evidenc* adj1 base*).ti,ab.

180027

216

best-practic*.ti,ab.

38823

217

(decision* adj2 mak*).ti,ab.

238310

218

(health* adj1 equit*).ti,ab.

8639

219

coordinat*.ti,ab.

331653

220

data.ti,ab.

4848912

221

information*.ti,ab.

1586879

222

or/173-221

10555284

223

exp Africa/

330180

224

africa.ti,ab.

146532

225

african countr*.ti,ab.

11952

226

AFRO.ti,ab.

3115

227

Algeria.ti,ab.

4222

228

(Djibouti or French Somaliland).ti,ab.

465

229

Egypt.ti,ab.

18463

230

Morocco.ti,ab.

7394

231

Tunisia.ti,ab.

8119

232

Libya.ti,ab.

1541

233

Angola.ti,ab.

1777

234

(Cameroon or Kamerun or Cameroun).ti,ab.

8790

235

(Cape Verde or Cabo Verde).ti,ab.

787

236

(Comoros or Glorioso Islands or Mayotte).ti,ab.

741

237

(Congo not ((Democratic Republic adj3 Congo) or congo red)).ti,ab.

4922

238

(Cote d'Ivoire or Cote dIvoire or Ivory Coast).ti,ab.

4583

239

(eSwatini or Swaziland).ti,ab.

1203

240

(Ghana or Gold Coast).ti,ab.

15654

241

(Kenya or East Africa Protectorate).ti,ab.

23155

242

(Lesotho or Basutoland).ti,ab.

978

243

Mauritania.ti,ab.

756

244

Nigeria.ti,ab.

36999

245

(Sao Tome adj2 Principe).ti,ab.

201

246

Senegal.ti,ab.

6664

247

Sudan.ti,ab.

9682

248

(Zambia or Northern Rhodesia).ti,ab.

6879

249

(Zimbabwe or Southern Rhodesia).ti,ab.

7015

250

(Botswana or Bechuanaland or Kalahari).ti,ab.

3298

251

(Equatorial Guinea or Spanish Guinea).ti,ab.

520

252

(Gabon or Gabonese Republic).ti,ab.

2056

253

(Mauritius or Agalega Islands).ti,ab.

1181

254

Namibia.ti,ab.

2069

255

(Benin or Dahomey).ti,ab.

4262

256

(Burkina Faso or Burkina Fasso or Upper Volta).ti,ab.

5424

257

(Burundi or Ruanda-Urundi).ti,ab.

1156

258

(Central African Republic or Ubangi-Shari).ti,ab.

1193

259

Chad.ti,ab.

1528

260

(((Democratic Republic or DR) adj2 Congo) or Congo-Kinshasa or Belgian Congo or Zaire or Congo Free State).ti,ab.

5540

261

Eritrea.ti,ab.

721

262

(Ethiopia or Abyssinia).ti,ab.

28331

263

Gambia.ti,ab.

2780

264

(Guinea not (New Guinea or Guinea Pig* or Guinea Fowl or Guinea-Bissau or Portuguese Guinea or Equatorial Guinea)).ti,ab.

3329

265

(Guinea-Bissau or Portuguese Guinea).ti,ab.

1193

266

Liberia.ti,ab.

1940

267

(Madagascar or Malagasy Republic).ti,ab.

5752

268

(Malawi or Nyasaland).ti,ab.

9175

269

Mali.ti,ab.

4437

270

(Mozambique or Mocambique).ti,ab.

4700

271

(Niger not (Aspergillus or Peptococcus or Schizothorax or Cruciferae or Gobius or Lasius or Agelastes or Melanosuchus or radish or Parastromateus or Orius or Apergillus or Parastromateus or Stomoxys)).ti,ab.

4161

272

(Rwanda or Ruanda).ti,ab.

4280

273

(Sierra Leone or Salone).ti,ab.

2949

274

(Somalia or Somaliland).ti,ab.

2041

275

South Sudan.ti,ab.

810

276

(Tanzania or Tanganyika or Zanzibar).ti,ab.

17663

277

(Togo or Togolese Republic or Togoland).ti,ab.

1825

278

Uganda.ti,ab.

19386

279

Seychelles/

451

280

Seychelles.ti,ab.

901

281

Sahel.ti,ab.

921

282

Reunion/

679

283

(reunion adj1 island).ti,ab.

990

284

or/223-283

467338

285

139 and 172 and 222 and 284

3329

286

limit 285 to yr="2010 -Current"

2631

287

remove duplicates from 286

2619

## Appendix 2: Conceptual framework

**Pandemic Preparedness Framework for Climate Sensitive Infectious Disease in Africa. Adapted from: World Health Organization. (2014). Ebola and Marburg virus disease epidemics: Preparedness, alert, control, and evaluation.** [**https://www.who.int/publications-detail-redirect/WHO-HSE-PED-CED-2014.05**](https://www.who.int/publications-detail-redirect/WHO-HSE-PED-CED-2014.05)

## Appendix 3: Topic mapping of studies to conceptual framework

**Percentage of data system or economic studies mapped to stages of pandemic preparedness. Studies could be relevant to all four stages; percentage was calculated as tagged stage by total of tagged stages in data system studies (n = 50) or economic studies (n = 18).**
